# The protease ADAMTS5 controls ovarian cancer cell invasion, downstream of Rab25

**DOI:** 10.1111/febs.70080

**Published:** 2025-03-31

**Authors:** Shengnan Yuan, Rachele Bacchetti, Jamie Adams, Doretta Cuffaro, Armando Rossello, Elisa Nuti, Salvatore Santamaria, Elena Rainero

**Affiliations:** ^1^ School of Biosciences University of Sheffield UK; ^2^ Clinical Medicine, School of Medicine & Population Health University of Sheffield UK; ^3^ Department of Pharmacy University of Pisa Italy; ^4^ Department of Biochemical Sciences, School of Biosciences, Faculty of Health and Medical Sciences University of Surrey Guildford UK

**Keywords:** ADAMTS5, cancer cell invasion, ECM, ovarian cancer, Rab25

## Abstract

Ovarian cancer is the 3rd most common gynaecological malignancy worldwide, with a 5‐year survival rate of < 30% in the presence of metastasis. Metastatic progression is characterised by extensive remodelling of the extracellular matrix, primarily mediated by secreted proteases, including members of the ‘a disintegrin and metalloprotease with thrombospondin motif’ (ADAMTS) family. In particular, ADAMTS5 has been reported to be upregulated in ovarian malignant tumours compared to borderline and benign lesions, suggesting it might play a role in metastatic progression. Furthermore, it has been suggested that Rab25, a small GTPase of the Ras family, might upregulate ADAMTS5 expression in ovarian cancer cells. Here we demonstrated that Rab25 promotes ADAMTS5 expression through the activation of the nuclear factor κB (NF‐κB) signalling pathway. Furthermore, ADAMTS5 was necessary and sufficient to stimulate ovarian cancer cell migration through complex fibroblast‐secreted matrices, while selective ADAMTS5 inhibition prevented ovarian cancer spheroid invasion in 3D systems. Finally, in ovarian cancer patients, high ADAMTS5 expression correlated with poor prognosis. Altogether, these data identify ADAMTS5 as a novel regulator of ovarian cancer cell migration and invasion, suggesting it might represent a previously undescribed therapeutic target to prevent ovarian cancer metastasis.

## Graphical abstract

Ovarian cancer is a common and often fatal gynaecological malignancy. Metastatic progression is characterised by extensive remodelling of the extracellular matrix, primarily mediated by secreted proteases, including ADAMTS family members. We found that the small GTPase Rab25 promoted the expression of ADAMTS5 in ovarian cancer cells, through the activation of the NF‐κB signalling pathway. ADAMTS5 was necessary and sufficient to drive invasive cell migration, in both 2D and 3D systems. Importantly, selected ADAMTS5 inhibition prevented cell migration. Thus, ADAMTS5 is a novel regulator of ovarian cancer cell migration and invasion, suggesting it might represent a previously undescribed therapeutic target.

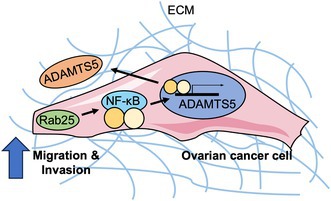

**Abbreviations**
ADAMTS: a disintegrin and metalloprotease with thrombospondin motif
CAF: cancer‐associated fibroblast
ECM: extracellular matrix
EGF: epidermal growth factor
ERK: extracellular signal‐regulated kinase
HIF: hypoxia inducible factor
MAPK: mitogen activated protein kinase
NF‐κB: nuclear factor κB
OC: ovarian cancer
PI3K: phosphatidylinositol 3 kinase
TIF: telomerase‐immortalised fibroblast
TME: tumour microenvironment
TNFα: tumour necrosis factor α

## Introduction

Ovarian cancer (OC) is the 3rd lethal gynaecological cancer worldwide [1]. Patients' deaths are primarily due to late‐stage diagnosis, metastasis, and chemotherapy resistance [2]. While the 5‐year survival rate of OC patients diagnosed at the early stages is over 70%, this drops to < 30% in the presence of peritoneal or distant metastasis [3]. The peritoneum represents the primary metastatic site, and metastatic colonisation is characterised by extensive remodelling of the extracellular matrix (ECM), a dynamic network of secreted proteins responsible for the biomechanical properties of the tissue.

The tumour microenvironment (TME) and the ECM play a key role in promoting cancer development and metastasis [4]. The ECM is continuously remodelled by ECM‐modifying enzymes, including matrix metalloproteinases, secreted by both cancer cells and cancer‐associated fibroblasts (CAFs) [5]. Indeed, altered ECM remodelling, caused by deregulated expression of ECM‐modifying enzymes, was found to promote the proliferation, migration, and invasion of tumour cells from different cancer types, including OC [6].

A disintegrin and metalloprotease with thrombospondin motif (ADAMTS) is a family of 19 secreted zinc‐dependent metalloproteases [7], the expression of which has been reported to be dysregulated in multiple cancer types [8]. In OC, ADAMTS5 expression was found significantly increased in malignant tumour samples compared to borderline and benign tumours, suggesting that ADAMTS5 could promote OC metastasis [9]. However, the role of ADAMTS5 in controlling OC cell migration and invasion is still unclear. Interestingly, ADAMTS5 has been suggested to be upregulated in OC cells over‐expressing Rab25 [10], a small GTPase of the RAS superfamily [11], whose expression was found significantly upregulated in advanced OC stages [12]. At the molecular level, Rab25 promoted OC cell migration and invasion in 3D matrices by enhancing the recycling of the ECM receptor integrin α5β1 at the pseudopod tips of OC cells [13].

Here we demonstrated that Rab25 promoted the expression of ADAMTS5 in a nuclear factor κB (NF‐κB)‐dependent manner. Importantly, secreted ADAMTS5 was necessary and sufficient to drive OC cell migration through fibroblast‐generated 3D matrices in a proteolytic activity‐dependent manner. Furthermore, Rab25 and ADAMTS5 downregulation opposed CAF‐driven OC cell spheroid invasion in 3D systems without affecting cell proliferation. Finally, elevated ADAMTS5 expression correlated with poor prognosis in OC patients. Altogether, these data indicate that ADAMTS5 could represent a novel druggable therapeutic target to prevent OC migration and invasion.

## Results

### Rab25 induced ADAMTS5 expression in OC cells

**Fig. 1:**
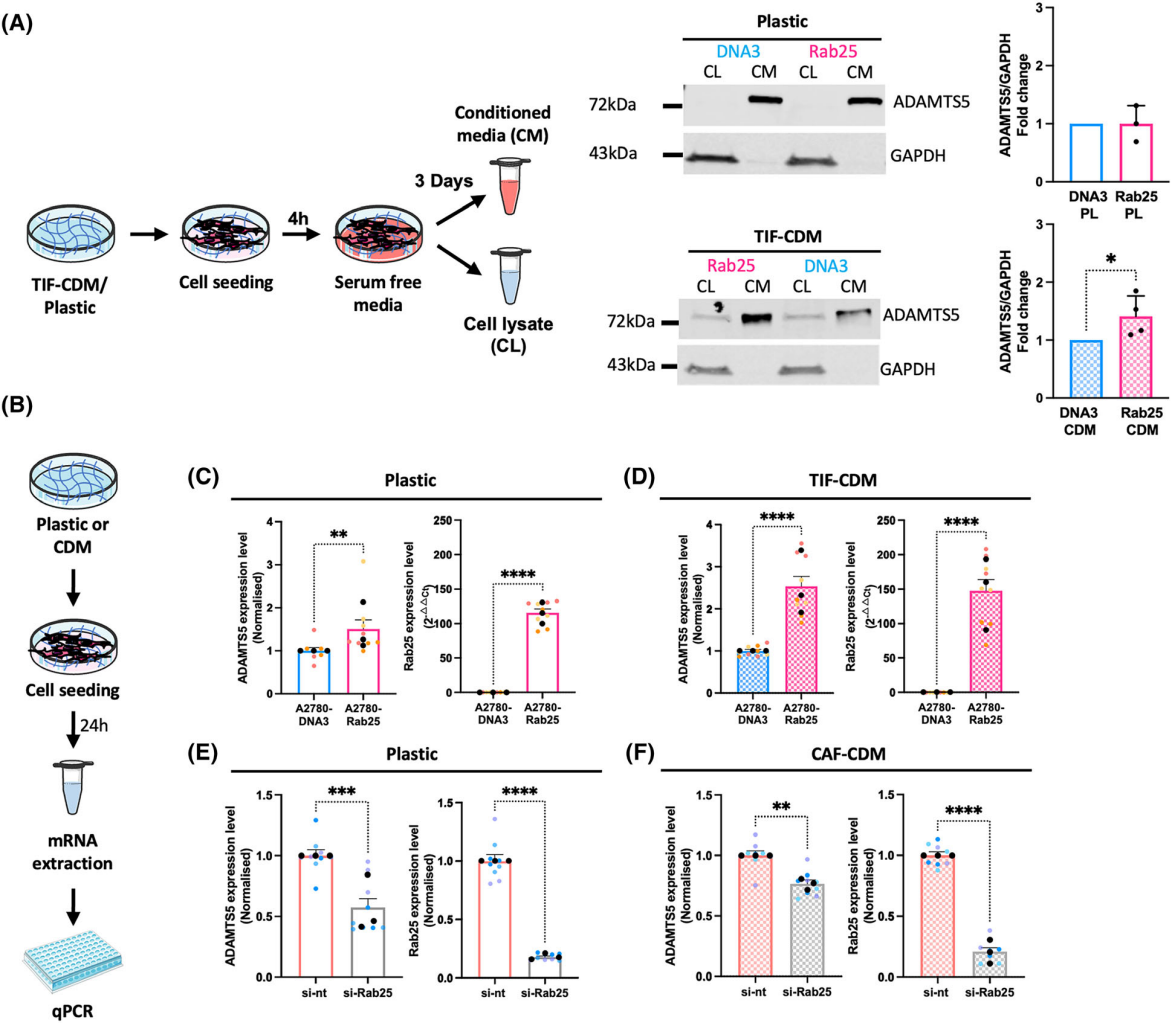
Rab25 induced ADAMTS5 expression in OC cells. (A) Schematic, cell lysate (CL) and conditioned media (CM) harvesting. A2780‐DNA3 and A2780‐Rab25 cells were plated on plastic (PL, *N* = 3 independent experiments) or telomerase‐immortalised fibroblasts (TIF) cell‐derived matrix (CDM, *N* = 4 independent experiments), cell lysates and concentrated conditioned media were extracted, protein levels of ADAMTS5 in CL and CM, and GAPDH in CL were measured by Western Blotting. Membranes were imaged with a Licor Odyssey Sa system, and the band intensity was quantified by image studio lite software. Data are presented as mean ± SEM. *P = 0.0286, Mann–Whitney test. (B) Schematic of the experimental plan. (C, D) A2780‐DNA3 and A2780‐Rab25 cells were seeded on plastic (C) or TIF‐CDM (D) and the mRNA was extracted and quantified by qPCR. The data were normalised to A2780‐DNA3 cells (ADAMTS5) or plotted in 2^−∆∆Ct^ (Rab25). Data are mean ± SEM from *N* = 3 independent experiments. The black dots represent the mean of individual experiments. **P = 0.005, ****P < 0.0001, Mann–Whitney test. (E, F) OVCAR3 cells were transfected with a non‐targeting siRNA control (si‐nt) or a Rab25‐targeting siRNA (si‐Rab25) and seeded on plastic (E) or CAF‐CDM (F). ADAMTS5 and Rab25 mRNA levels were quantified by qPCR and normalised to si‐nt. Data are presented as mean ± SEM from *N* = 3 independent experiments. The black dots represent the mean of individual experiments. **P = 0.0012, ***P = 0.0003, ****P < 0.0001, Mann–Whitney test.

ADAMTS5 was identified in a microarray screen to detect ECM‐ and Rab25‐dependent changes in gene expression in OC cells [10]. To validate the role of Rab25 in inducing ADAMTS5 expression, A2780 cells stably expressing Rab25 (A2780‐Rab25) or empty vector pcDNA3 controls (A2780‐DNA3) were seeded on plastic or cell‐derived matrix generated by telomerase immortalised fibroblasts (TIF‐CDM), a 3D matrix rich in collagen and fibronectin that recapitulates multiple features of ECM *in vivo* [14], and the protein levels of ADAMTS5 were measured by western blotting. Since ADAMTS5 is a secreted protease, we harvested conditioned media from A2780‐DNA3 and A2780‐Rab25 cells and found that ADAMTS5 levels were significantly increased in A2780‐Rab25 cells seeded on CDM, while there was no significant difference in ADAMTS5 levels between A2780‐DNA3 and A2780‐Rab25 cells seeded on plastic (Fig. 1A). ADAMTS5 levels have been shown to be modulated by low‐density lipoprotein receptor‐related protein 1 (LRP1)‐mediated endocytosis in chondrocytes [15]; however, we did not detect any changes in LRP1 expression in A2780‐DNA3 and A2780‐Rab25 cells (Fig. S1). To determine whether this change occurred at the mRNA level, we assessed ADAMTS5 expression by RT‐qPCR (Fig. 1B). Remarkably, we detected a 2.5‐fold increase in ADAMTS5 mRNA levels in A2780‐Rab25 cells compared to A2780‐DNA3 cells on CDM (Fig. 1D), while a smaller but still statistically significant increase (1.5‐fold) was measured on plastic (Fig. 1C). These data indicate that Rab25 increased ADAMTS5 levels and the presence of ECM further enhanced Rab25‐dependent ADAMTS5 expression in OC cells.

To study the role of Rab25 in a more physiological context, we measured endogenous Rab25 expression in a panel of OC cell lines and found that OVCAR3 cells, but not OVCAR4 and SKOV3, overexpressed Rab25, to a similar level to the exogenous overexpression in A2780 cells (Fig. S2). Western blot analysis showed that Rab25 ran at different molecular weights in the two cell lines, probably because the Rab25 construct overexpressed in A2780 cells is tagged. Even though the HA tag is only nine amino acids long (1.1 kDa), it might affect the way the protein runs in the gel. OVCAR3 cells were transfected with a non‐targeting siRNA control or an siRNA targeting Rab25 and ADAMTS5 expression was assessed by RT‐qPCR in cells seeded on plastic or CDM generated by omental CAFs [16]. Consistent with our previous data, the downregulation of Rab25 significantly reduced ADAMTS5 mRNA levels in OVCAR3 cells, both on plastic and on CDM, although here there was a more prominent effect on plastic (Fig. 1E,F). These data demonstrate that Rab25 induced ADAMTS5 expression in OC cells, while the ECM had a more prominent role in A2780 compared to OVCAR3 cells.

### The transcription factor NF‐κB was required for Rab25‐induced ADAMTS5 expression in OC cells

**Fig. 2:**
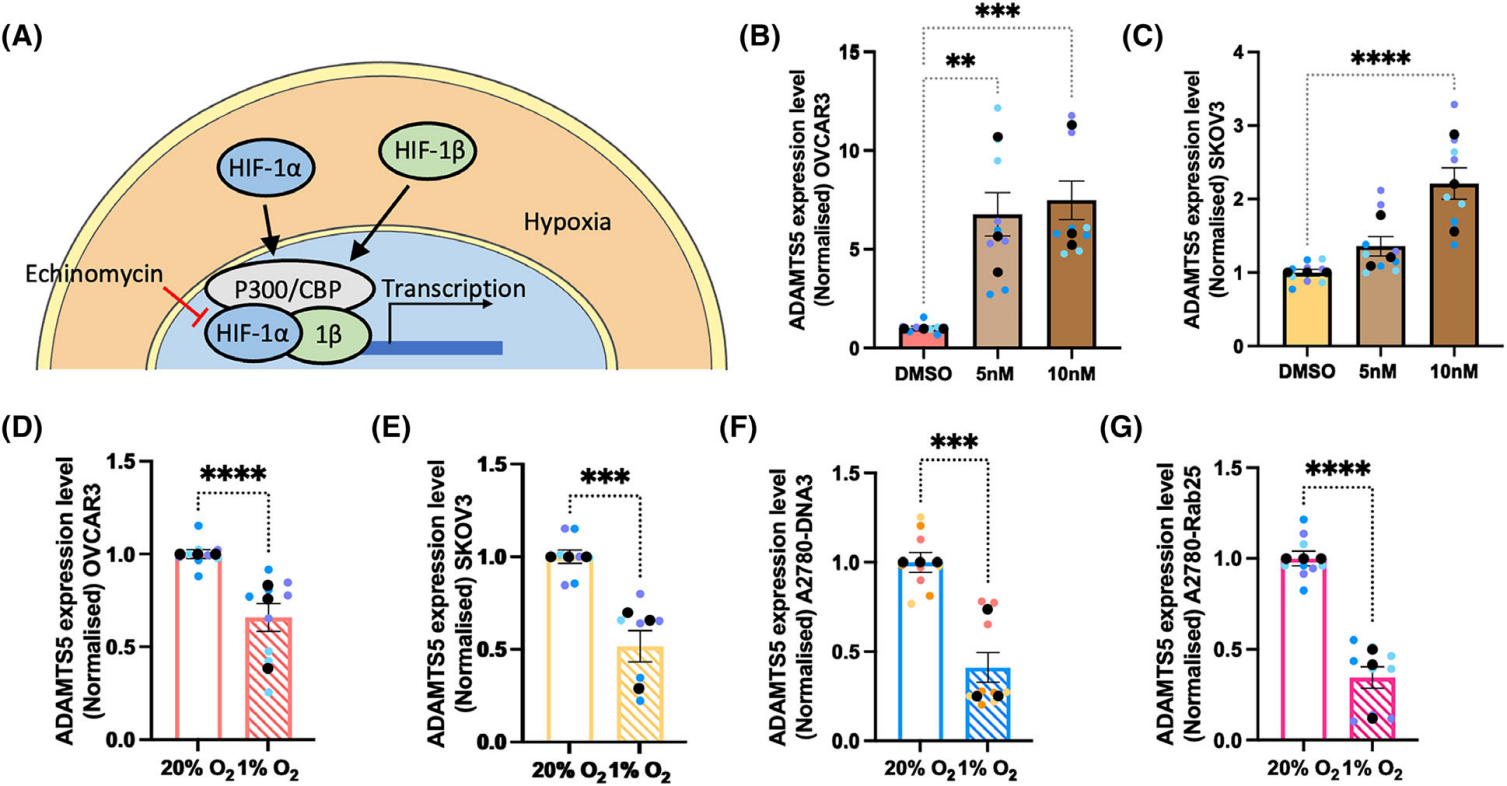
HIF‐1 suppressed ADAMTS5 expression in OC cells. (A) Schematic, HIF‐1 signalling pathway. (B, C) OVCAR3 (B) and SKOV3 (C) cells were treated with DMSO, 5 or 10 nm Echinomycin for 24 h, and the mRNA levels of ADAMTS5 were measured by qPCR. Data were normalised to the DMSO control and presented as mean ± SEM from *N* = 3 independent experiments. The black dots represent the mean of individual experiments. **P = 0.0011, ***P = 0.0003, ****P < 0.0001, Kruskal–Wallis test. (D–G) OVCAR3 (D), SKOV3 (E), A2780‐DNA3 (F) and A2780‐Rab25 (G) cells were incubated under Normoxia (20% O_2_) or Hypoxia (1% O_2_) for 24 h, and the mRNA levels of ADAMTS5 were measured by qPCR. Data were normalised to Normoxia control and presented as mean ± SEM from *N* = 3 independent experiments. The black dots represent the mean of individual experiments. ***P < 0.001, ****P < 0.0001, Mann–Whitney test.

Having established that Rab25 promoted ADAMTS5 expression, we set out to characterise the transcription factor(s) responsible for this. Rab25 has been reported to stabilise hypoxia inducible factor 1α (HIF1α) protein levels in normoxia in OC cells [17]. Since HIF1α was reported to weakly bind to the ADAMTS5 promoter [18], we investigated the role of HIF1 in ADAMTS5 expression by treating the cells with a well‐characterised HIF inhibitor, echinomycin [19], or incubating the cells under hypoxia for 24 h. Surprisingly, echinomycin treatment significantly increased ADAMTS5 expression in both OVCAR3, endogenously over‐expressing Rab25, and in SKOV3 cells, which lack detectable Rab25 expression (Fig. 2A–C). Consistently, hypoxia incubation significantly reduced ADAMTS5 expression in all the cell lines tested, regardless of the expression of Rab25 (Fig. 2D–G). Together, these data indicate that HIF1 is a negative regulator of ADAMTS5 expression and is not involved in Rab25‐dependent ADAMTS5 expression in OC cells.

**Fig. 3:**
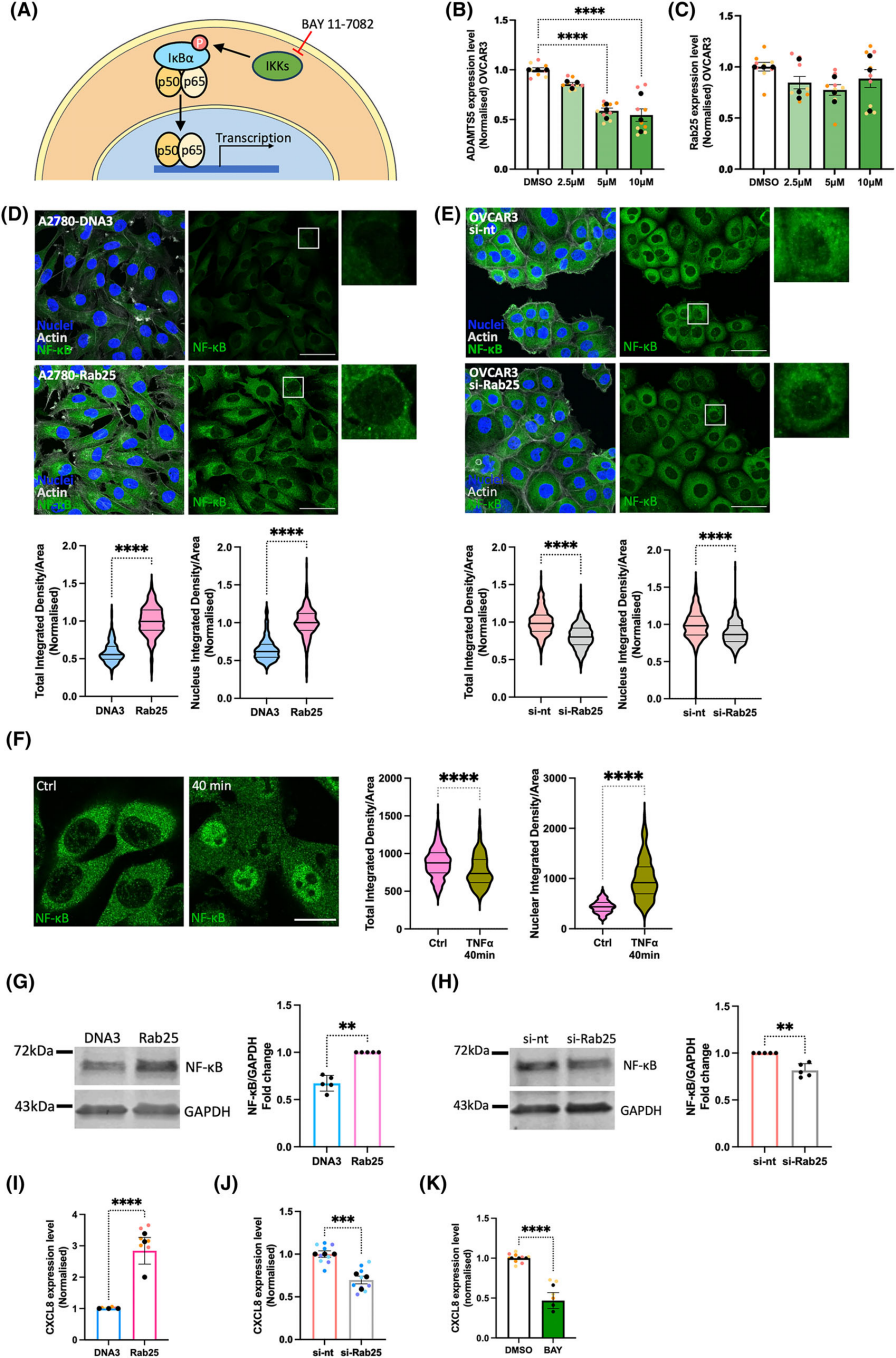
Rab25 induced ADAMTS5 expression in an NF‐κB dependent manner. (A) Schematic, NF‐κB signalling pathway. (B, C) OVCAR3 cells were treated with DMSO, 2.5, 5 or 10 μm BAY 11‐7082 for 24 h and the mRNA levels of ADAMTS5 (B) and Rab25 (C) were measured by qPCR. Data were normalised to the DMSO control and presented as mean ± SEM from *N* = 3 independent experiments. The black dots represent the mean of individual experiments. ****P < 0.0001, Kruskal–Wallis test. (D, E) A2780‐DNA3, A2780‐Rab25 cells (D), OVCAR3 cells transfected with non‐targeting (si‐nt) or Rab25 targeting (si‐Rab25) si‐RNA (E) were seeded on glass‐bottom dishes, fixed, stained for NF‐κB (green), nuclei (blue) and Actin (grey), and imaged with a Nikon A1 confocal microscope. Scale bar, 50 μm. NF‐κB integrated density for the whole cell and the nucleus was measured with imagej and normalised to the cell area. Data were plotted as violin plots (median and quartiles) from *N* = 3 independent experiments. ****P < 0.0001, Mann–Whitney test. (F) A2780‐Rab25 were stimulated with 40 ng·mL^−1^ TNFα for 40 min, fixed, stained for NF‐κB (green), Actin and nuclei and imaged with a Nikon A1 confocal microscope. Scale bar, 20 μm. NF‐κB integrated density for the whole cell and the nucleus was measured with imagej and normalised to the cell area. Data were plotted as violin plots (median and quartiles) from *N* = 3 independent experiments. ****P < 0.0001, Mann–Whitney test. (G, H) NF‐κB and GAPDH protein levels in A2780‐DNA3 and A2780‐Rab25 cells (G) or OVCAR3 cells transfected with non‐targeting (si‐nt) or Rab25 targeting (si‐Rab25) si‐RNA (H) were quantified by Western Blotting. Membranes were imaged with a Licor Odyssey Sa system, and the band intensity was quantified by image studio lite software. The fold change of NF‐κB/GAPDH was plotted. Data are presented as mean ± SEM from *N* = 5 independent experiments. **P = 0.0079 for both G and H, Mann–Whitney test. (I) CXCL8 mRNA levels in A2780‐DNA3 and Rab25 cells were quantified by qPCR and normalised to A2780‐DNA3 cells. Data are presented as mean ± SEM from *N* = 3 independent experiments. ****P < 0.0001, Mann–Whitney test. (J) CXCL8 mRNA levels in OVCAR3 cells transfected with non‐targeting (si‐nt) or Rab25 targeting (si‐Rab25) si‐RNA were quantified by qPCR and normalised to si‐nt. Data are presented as mean ± SEM from *N* = 3 independent experiments. ***P < 0.001, Mann–Whitney test. (K) CXCL8 mRNA levels in OVCAR3 cells treated with DMSO or 10 μm BAY 11‐7082 for 24 h were quantified by qPCR and normalised to DMSO control. Data are presented as mean ± SEM from *N* = 3 independent experiments. ****P < 0.0001, Mann–Whitney test.

ADAMTS5 has been extensively studied in osteoarthritis, where its catalytic activity has been shown to contribute to the disease progression [20]. In chondrocytes, a subunit of transcription factor NF‐κB, RelA/p65, was found to interact with the ADAMTS5 promoter and strongly induce ADAMTS5 expression [18]. Therefore, we tested whether NF‐κB also promoted ADAMTS5 expression in OC cells. BAY 11‐7082 is a small molecule inhibitor specifically targeting IKK (inhibitor of nuclear factor kappa B kinase), which prevents the phosphorylation and degradation of IκBα (inhibitor of nuclear factor kappa B). As a result of BAY 11‐7082 treatment, the release, nuclear translocation, and promoter binding of NF‐κB are inhibited (Fig. 3A) [21]. OVCAR3 cells were treated with DMSO, 2.5, 5, or 10 μm BAY 11‐7082 for 24 h, and the expression of ADAMTS5 was quantified by RT‐qPCR. As a result, ADAMTS5 expression decreased in a dose‐dependent manner, to a similar extent to the reduction we observed upon Rab25 knock‐down (Fig. 3B). Importantly, Rab25 expression was not affected by BAY 11‐7082 treatment (Fig. 3C), indicating that NF‐κB regulated ADAMTS5 expression downstream of Rab25.

To elucidate whether Rab25 controlled NF‐κB signalling, we assessed the intracellular localisation of the NF‐κB subunit RelA/p65 in A2780‐DNA3 and A2780‐Rab25 cells. RelA/p65 staining was predominately cytoplasmic, with weak nuclear staining. RelA/p65 signal intensity was significantly increased within the whole cells and in the nuclei of Rab25‐overexpressing A2780 cells compared to DNA3 cells (Fig. 3D). Similarly, Rab25 knockdown in OVCAR3 cells resulted in a small but statistically significant reduction in RelA/p65 overall and nuclear intensity, in comparison to the non‐targeting control (Fig. 3E). Tumour necrosis factor α (TNFα) is a well‐established promoter of NF‐κB activation [22]. Indeed, TNFα stimulation resulted in a strong NF‐κB nuclear translocation (Fig. 3F). Consistently, increased NF‐κB protein levels were detected by western blotting in Rab25‐overexpressing A2780 cells compared to DNA3 control cells (Fig. 3G), while Rab25 knockdown resulted in a small but statistically significant reduction in NF‐κB protein levels in OVCAR3 cells (Fig. 3G,H). To confirm the role of Rab25 in promoting NF‐κB activation, we measured the expression of CXCL8, a canonical NF‐κB target gene encoding IL‐8 [23]. Consistently, Rab25 overexpression in A2780 cells significantly increased CXCL8 mRNA levels (Fig. 3I), while Rab25 downregulation significantly reduced CXCL8 expression in OVCAR3 cells (Fig. 3J). Consistent with the fact that CXCL8 is an NF‐κB target gene, treatment with BAY 11‐7082 resulted in a significant reduction in CXCL8 mRNA levels (Fig. 3K). Taken together, these results suggest that Rab25 induced ADAMTS5 expression through the regulation of NF‐κB activation.

**Fig. 4:**
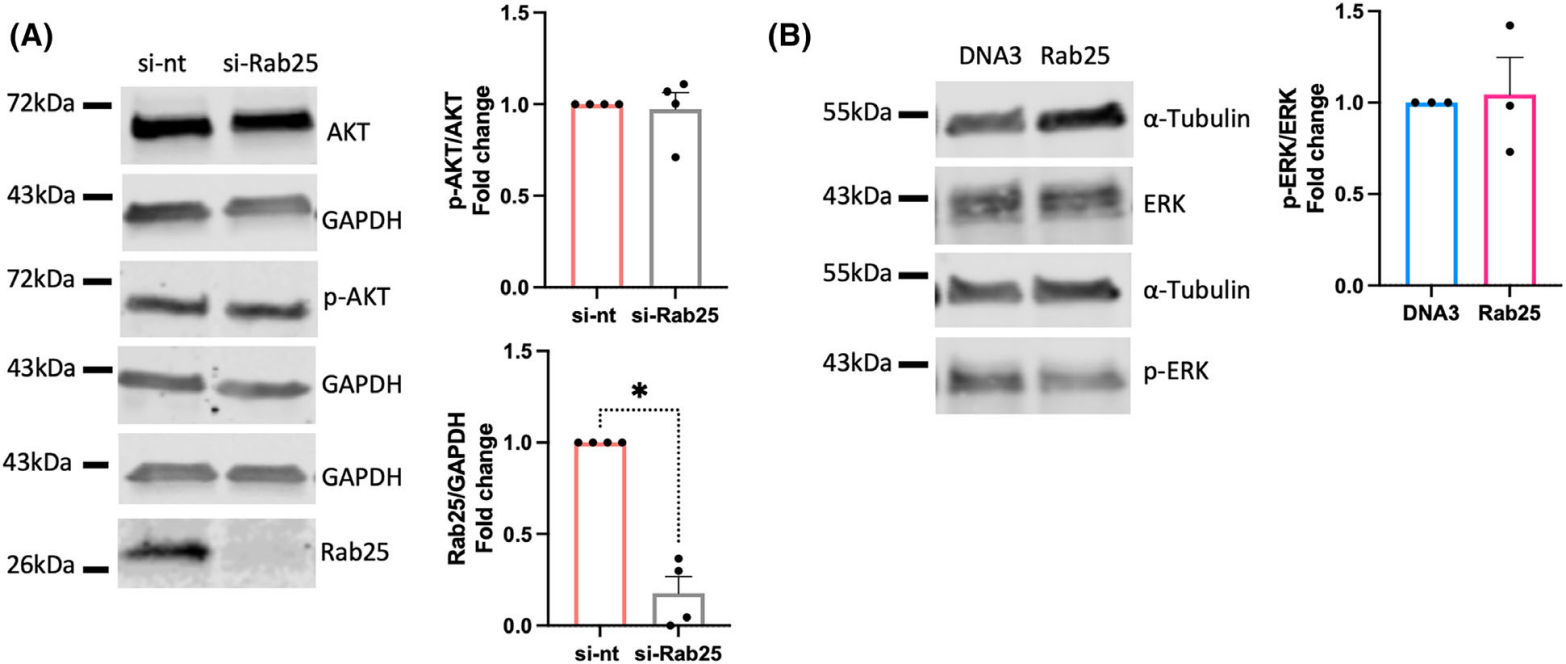
Rab25 downregulation did not affect AKT nor ERK phosphorylation. (A) OVCAR3 cells were transfected with a non‐targeting siRNA control (si‐nt) or Rab25 targeting siRNA (si‐Rab25) and the protein levels of AKT, p‐AKT, Rab25, and GAPDH were quantified by Western Blotting. Membranes were imaged with a Licor Odyssey Sa system, and the band intensity was quantified by image studio lite software. The fold change of normalised p‐AKT/AKT and Rab25/GAPDH was plotted. Data are presented as mean ± SEM from *N* = 4 independent experiments. *P = 0.0286, Mann–Whitney test. (B) The protein levels of ERK, p‐ERK, and α‐Tubulin in A2780‐DNA3 and A2780‐Rab25 cells were quantified by Western blotting as in A. The fold change of normalised p‐ERK/ERK was plotted. Data are presented as mean ± SEM from *N* = 3 independent experiments. Mann–Whitney test.

Several signalling pathways have been shown to control NF‐κB. For instance, epidermal growth factor (EGF)‐mediated EGFR stimulation was found to promote NF‐κB activation via phosphatidylinositol 3 kinase (PI3K)/AKT and mitogen‐activated protein kinase (MAPK)/extracellular signal‐regulated kinase (ERK) pathways in OC cells [24]. Interestingly, Rab25 downregulation was found to impair EGFR signalling, with a consequent reduction in ERK and AKT phosphorylation in radioresistant lung adenocarcinoma and nasopharyngeal carcinoma cells [25]. We therefore investigated whether Rab25 controlled these signalling pathways in OC cells. However, Rab25 overexpression in A2780 cells [26] or Rab25 knockdown in OVCAR3 cells did not affect AKT phosphorylation (Fig. 4A). Similarly, Rab25 overexpression did not affect ERK phosphorylation in A2780 cells (Fig. 4B), suggesting that Rab25 controlled NF‐κB through an AKT‐ and ERK‐independent signalling pathway.

### ADAMTS5 was required for Rab25‐dependent OC cell migration on CDM

**Fig. 5:**
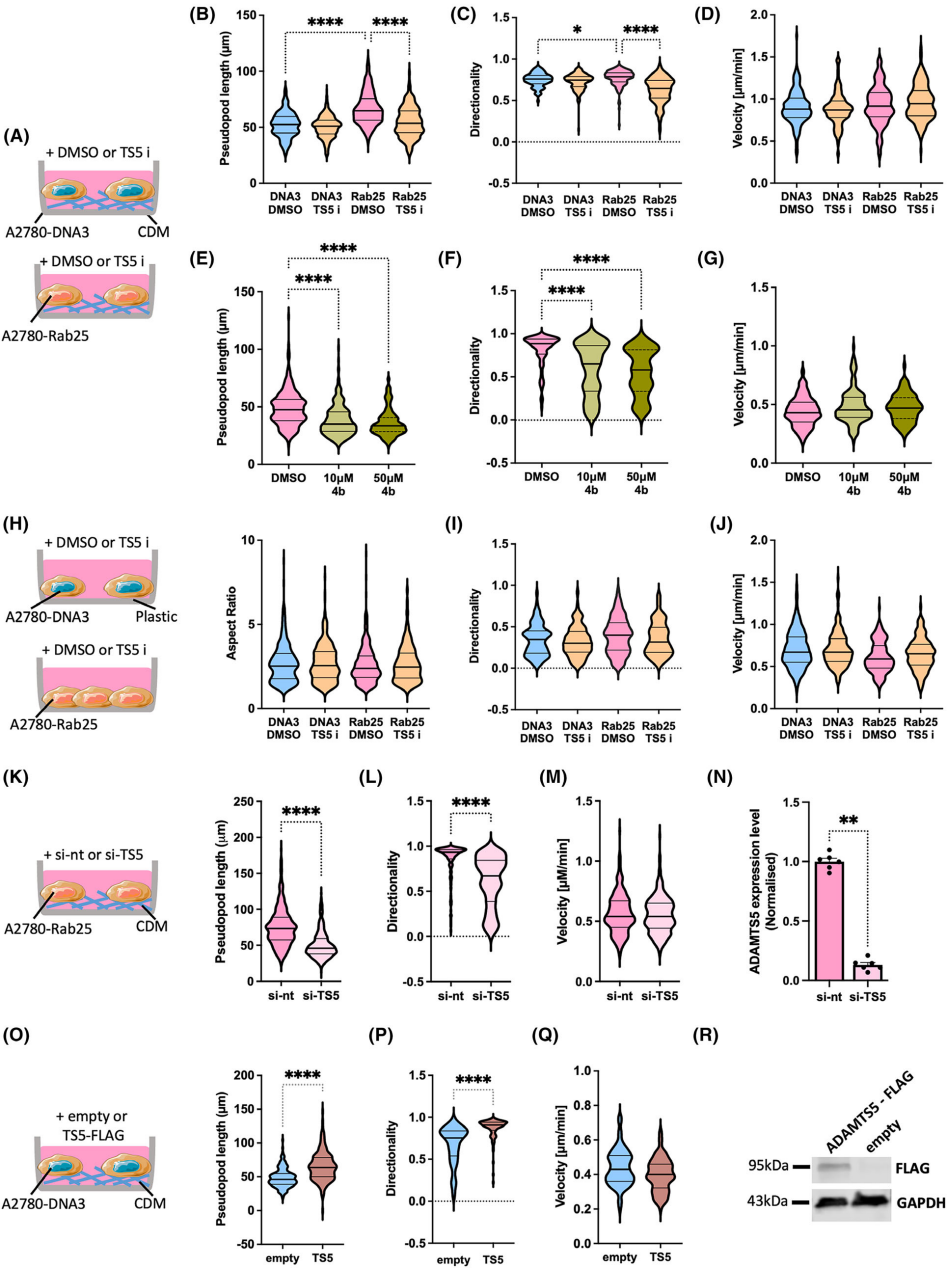
ADAMTS5 was required for Rab25‐dependent pseudopod elongation and directional migration of OC cells on CDM. (A–G) A2780‐DNA3 and A2780‐Rab25 cells were seeded on CDM and treated with DMSO, 5 μm ADAMTS5 inhibitor (TS5 i), 10 or 50 μm 4b. Cells were imaged live with a 10× Nikon widefield live‐cell system (Nikon Ti eclipse with Oko‐lab environmental control chamber) for 16 h. The pseudopod length (μm) (B, E), directionality (C, F) and velocity (μm·min^−1^) (D, G) were measured with imagej. Data were plotted as violin plots (median and quartiles) from *N* = 3 independent experiments. *P = 0.0155, ****P < 0.0001, Kruskal–Wallis test. (H–J) A2780‐DNA3 and A2780‐Rab25 cells were seeded on plastic, treated, and imaged as in A. The aspect ratio, measured as cell length/cell width (H), directionality (I) and velocity (μm·min^−1^) (J) were measured with imagej. Data were plotted as violin plots (median and quartiles) from *N* = 2 independent experiments. (K–M) A2780‐Rab25 cells were transfected with non‐targeting siRNA control (si‐nt) or ADAMTS5 targeting si‐RNA (si‐TS5) and seeded on CDM. Cells were imaged as in A and the pseudopod length (μm) (K), directionality (L) and velocity (μm·min^−1^) (M) were measured with imagej. Data were plotted as violin plots (median and quartiles) from *N* = 3 independent experiments. ****P < 0.0001, Mann–Whitney test. (N) A2780‐Rab25 cells were transfected as in D and the mRNA levels of ADAMTS5 and GAPDH were measured by qPCR. Data were normalised to si‐nt and presented as mean ± SEM from *N* = 2 independent experiments. **P = 0.0022, Mann–Whitney test. (O–R) A2780 cells were transfected with an empty vector control (empty) or ADAMTS5‐FLAG and seeded on CDM. Cells were imaged as in A and the pseudopod length (μm) (O), directionality (P) and velocity (μm·min^−1^) (Q) were measured with imagej. Data were plotted as violin plots (median and quartiles) from *N* = 2 independent experiments. ****P < 0.0001, Mann–Whitney test. (R) ADAMTS5‐FLAG and GAPDH levels were measured by Western Blotting. Image created with items adapted from Servier Medical Art, licensed under CC BY 4.0.

Rab25 was previously shown to promote OC cell migration in 3D matrices by enhancing integrin α5β1 recycling at the pseudopod tips [13]. We therefore investigated whether ADAMTS5 played a role in Rab25‐dependent cell migration, by seeding A2780‐DNA3 and A2780‐Rab25 cells on CDM in the presence of DMSO control or 5 μm of a small molecule active‐site zinc‐chelating ADAMTS5 inhibitor. Consistent with previous data [13], Rab25 overexpression significantly increased pseudopod elongation and directionality of cell migration, without affecting migration velocity. Remarkably, inhibition of ADAMTS5 catalytic activity significantly suppressed pseudopod elongation and directional migration in Rab25 overexpressing cells, without changing the morphology and migration of A2780‐DNA3 cells (Fig. 5A–C). The velocity of cell migration was not affected by ADAMTS5 inhibition (Fig. 5D). 4b is a glycoconjugated arylsulfonamide recently described as a highly selective ADAMTS5 inhibitor [27]. Consistently, 10 and 50 μm 4b significantly impaired pseudopod elongation and directional cell migration in A280‐Rab25 cells seeded on CDM, without affecting the velocity of cell migration (Fig. 5E–G). Importantly, Rab25 overexpression and ADAMTS5 inhibition did not affect cell migration on plastic (Fig. 5H–J), indicating that Rab25 and ADAMTS5's role in controlling OC cell migration was ECM‐dependent. Similarly, ADAMTS5 knockdown in A2780‐Rab25 cells significantly reduced pseudopod length and directional cell migration on CDM (Fig. 5K–N). Furthermore, transient over‐expression of ADAMTS5 promoted pseudopod elongation and directional cell migration in A2780 cells, in the absence of Rab25 over‐expression, compared to cells transfected with an empty vector control (Fig. 5O–R). Together, these data demonstrate that ADAMTS5 was required for Rab25‐dependent cell migration on CDM.

**Fig. 6:**
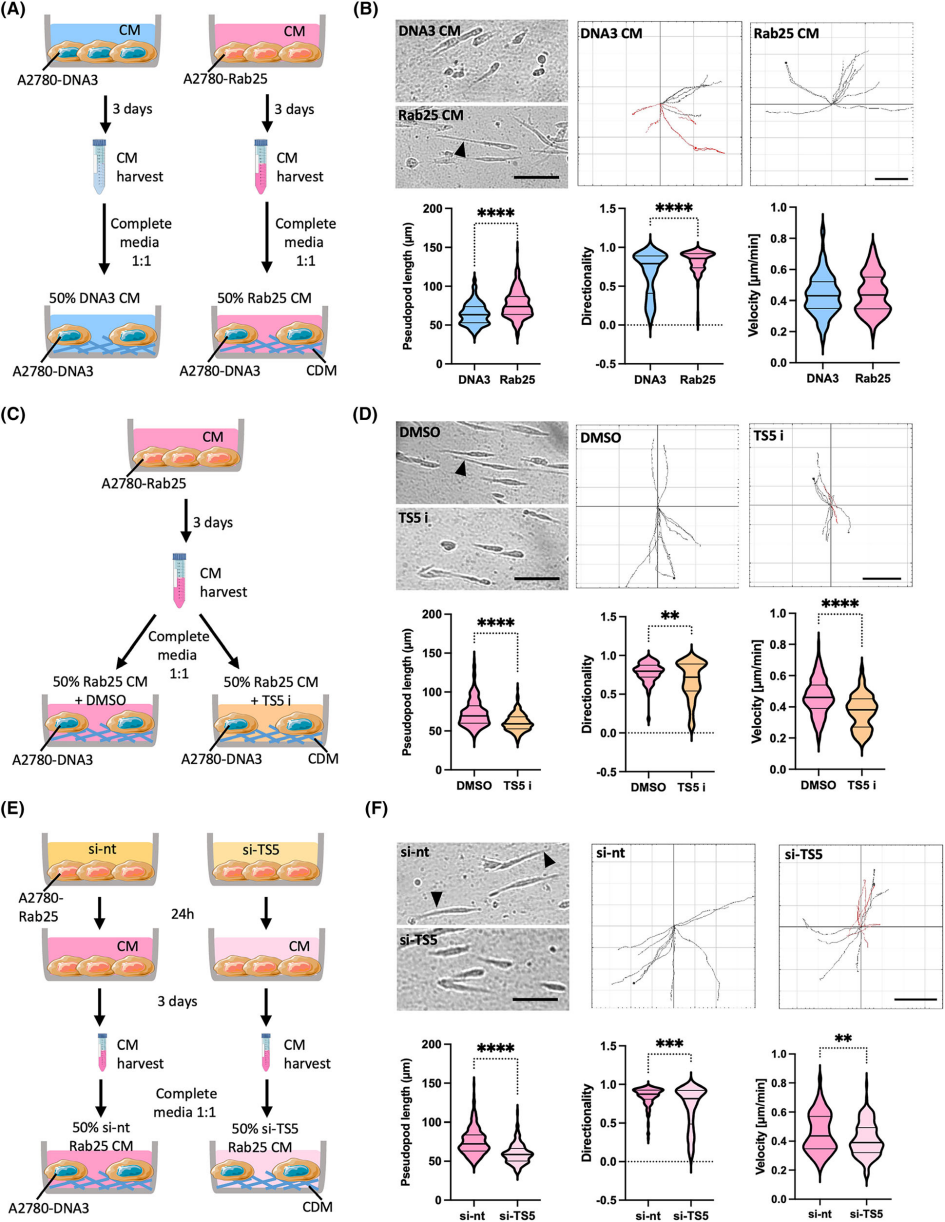
Conditioned media derived from A2780‐Rab25 cells enhanced pseudopod elongation and directional migration of A2780‐DNA3 cells on CDM, in an ADAMTS5‐dependent manner. (A, C, E) Schematics of the experimental workflow. Conditioned media (CM) was collected from A2780‐DNA3 and A2780‐Rab25 cells seeded on plastic for 3 days. (B, D) A2780‐DNA3 cells were seeded on CDM and treated with A2780‐DNA3 or Rab25 CM (diluted 1 : 1 in complete media) (B), or with Rab25 CM in the presence of DMSO control or 5 μm ADAMTS5 inhibitor (TS5 i, D). Cells were imaged live with a Nikon widefield live‐cell system (Nikon Ti eclipse with Oko‐lab environmental control chamber) for 16 h. Stills extracted from the movies are presented. The arrowheads point to the elongated pseudopods. Scale bar, 100 μm. Data were plotted as violin plots (median and quartiles) from *N* = 3 independent experiments. **P = 0.0057, ****P < 0.0001, Mann–Whitney test. (E, F) Conditioned media (CM) was collected from A2780‐Rab25 cells transfected with a non‐targeting siRNA control (si‐nt) or ADAMTS5 targeting si‐RNA (si‐TS5) and grown on plastic for 3 days. A2780‐DNA3 cells were seeded on CDM and treated with si‐nt or si‐TS5 A2780‐Rab25 CM (diluted 1 : 1 in complete media). Cells were imaged live with a Nikon widefield live‐cell system (Nikon Ti eclipse with Oko‐lab environmental control chamber) for 16 h. Stills extracted from the movies are presented. The arrowheads point to the elongated pseudopods. Scale bar, 100 μm. Representative spider plots show the migration paths of manually tracked cells (directionality > 0.5 in black, < 0.5 in red). Scale bar, 200 μm. The pseudopod length (μm), directionality and velocity (μm·min^−1^) of cell migration were measured with imagej. Data were plotted as violin plots (median and quartiles) from *N* = 3 independent experiments. **P = 0.0018, ***P = 0.0002, ****P < 0.0001, Mann–Whitney test. Image created with items adapted from Servier Medical Art, licensed under CC BY 4.0.

To more directly characterize the role of secreted ADAMTS5 in OC cell migration, we incubated A2780‐DNA3 cells with conditioned media generated by A2780‐Rab25 and DNA3 cells (Fig. 6A). Compared to A2780‐DNA3 conditioned media, treatment of A2780‐DNA3 cells with A2780‐Rab25 conditioned media significantly increased pseudopod length and directionality of cell migration, while the migration velocity was not affected (Fig. 6B). To confirm that ADAMTS5 catalytic activity was required for this, A2780‐DNA3 cells were treated with A2780‐Rab25 conditioned media in the presence of DMSO or 5 μm ADAMTS5 active site inhibitor, and their migration ability was measured (Fig. 6C). As a result, ADAMTS5 inhibition reduced the pseudopod length and migration directionality of A2780‐DNA3 cells treated with A2780‐Rab25 conditioned media (Fig. 6D). Interestingly, ADAMTS5 inhibition also reduced the migration velocity of A2780‐DNA3 cells, which was not identified in the absence of conditioned media (Fig. 5C). This might suggest a more effective target engagement by the inhibitor, as in the absence of conditioned media, ADAMTS5 might be mostly ECM‐bound and therefore more difficult to inhibit.

Several factors, other than ADAMTS5, could be present in Rab25‐overexpressing cell conditioned media and promote cell migration. Therefore, conditioned media was generated from control or ADAMTS5 knockdown A2780‐Rab25 cells and added to A2780‐DNA3 cells seeded on CDM (Fig. 6E). Consistent with the ADAMTS5 inhibition results, A2780‐DNA3 cells treated with conditioned media derived from ADAMTS5 knockdown A2780‐Rab25 cells showed a significantly lower pseudopod length, migration directionality, and velocity compared to conditioned media generated from non‐targeting siRNA treated cells (Fig. 6F). Altogether, these results demonstrate that secreted ADAMTS5 was sufficient to drive OC cells migration on CDM.

**Fig. 7:**
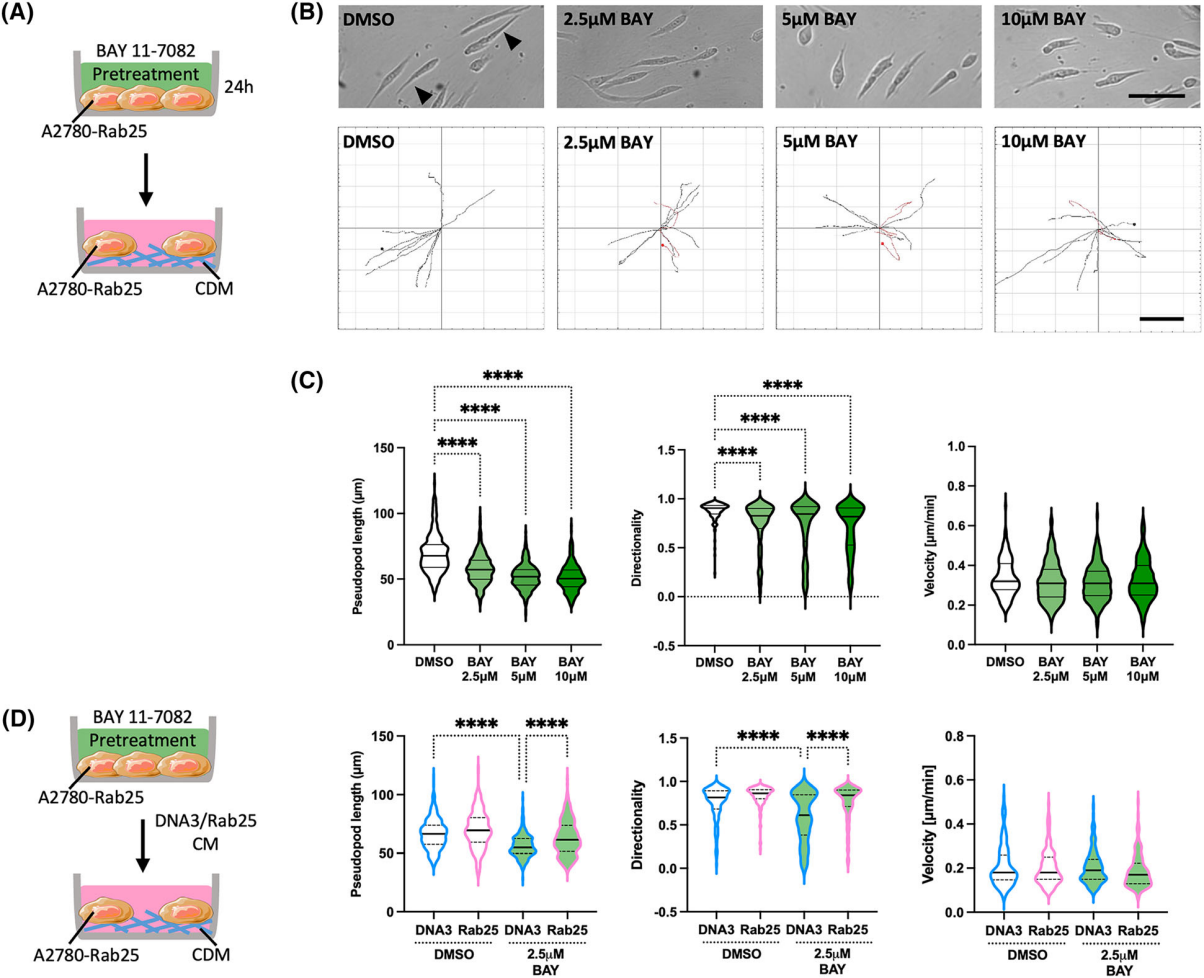
NF‐κB inhibition impaired A2780‐Rab25 cell migration on CDM. (A) Schematic of the experimental workflow. (B, C) A2780‐Rab25 cells were pretreated with DMSO, 2.5, 5, or 10 μm BAY 11‐7072 (BAY) for 24 h, seeded on CDM, and imaged live with a Nikon widefield live‐cell system (Nikon Ti eclipse with Oko‐lab environmental control chamber) for 16 h. Stills extracted from the movies are presented. The arrow‐heads point to the elongated pseudopods. Scale bar, 100 μm. Representative spider plots show the migration paths of manually tracked cells (directionality > 0.5 in black, < 0.5 in red). Scale bar, 200 μm. The pseudopod length (μm), directionality and velocity (μm·min^−1^) of cell migration were measured with imagej. Data were plotted as violin plots (median and quartiles) from *N* = 3 independent experiments. (D) A2780‐Rab25 cells were pretreated with DMSO or 2.5 μm BAY 11‐7072 (BAY) for 24 h, seeded on CDM, treated with conditioned media (CM) from A2780‐DNA3 (DNA3) or Rab25‐overexpressing A2780 cells (Rab25) and imaged live with a Nikon widefield live‐cell system (Nikon Ti eclipse with Oko‐lab environmental control chamber) for 16 h. Data were plotted as violin plots (median and quartiles) from *N* = 3 independent experiments. ****P < 0.0001, Kruskal–Wallis test. Image created with items adapted from Servier Medical Art, licensed under CC BY 4.0.

As NF‐κB was required for Rab25‐induced ADAMTS5 expression, we tested the effect of NF‐κB inhibition on the migration ability of A2780‐Rab25 cells. The cells were pretreated with DMSO or BAY 11‐7082 for 24 h, and cell migration on CDM was measured (Fig. 7A). Compared to the DMSO control, pretreatment with BAY 11‐7082 resulted in a dose‐dependent reduction in pseudopod length and migration directionally, while the velocity of cell migration was not affected (Fig. 7B,C). NF‐κB could modulate cell migration through several mechanisms. To assess whether, in this context, NF‐κB controlled cell migration through the regulation of ADAMTS5 expression, we performed conditioned media rescue experiments. A2780‐Rab25 cells were pretreated with BAY 11‐7082 and allowed to migrate on CDM in the presence of conditioned media generated from either control or Rab25‐overexpressing A2780 cells. Interestingly, we found that, while pretreatment with BAY 11‐7082 reduced both pseudopod elongation and directionality of cell migration in the presence of A2780‐DNA3 conditioned media, the presence of A2780‐Rab25 conditioned media fully rescued both pseudopod elongation and migration directionality (Fig. 7D). The velocity of cell migration was not affected by either BAY 11‐7082 or conditioned media treatment (Fig. 7D). These results show that NF‐κB signalling played a role in Rab25‐dependent migration of OC cells by promoting the expression of ADAMTS5.

### ADAMTS5 was required for Rab25‐dependent 3D invasion of OC cells

**Fig. 8:**
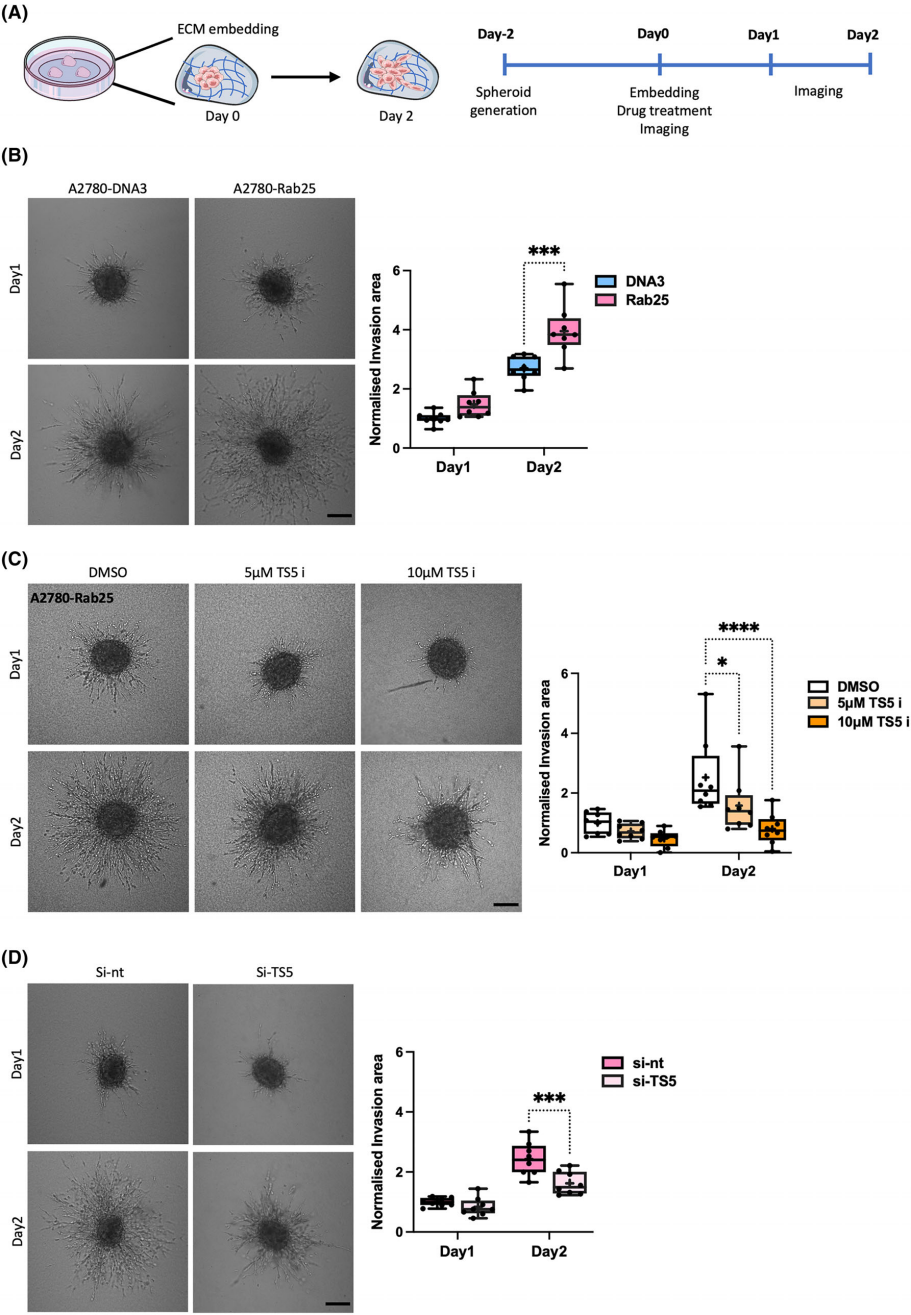
ADAMTS5 inhibition reduced the 3D invasion of OC cells. (A) Schematic of 3D invasion assays. (B) A2780‐DNA3 and A2780‐Rab25 spheroids generated by the hanging drop method were embedded in 3 mg·mL^−1^ Geltrex, 3 mg·mL^−1^ collagen I, and 25 μg·mL^−1^ fibronectin and imaged live with a Nikon A1 confocal microscope up to day 2. Scale bar, 200 μm. Spheroid invasion area was quantified with imagej and normalised to DNA3 day 1. Data are presented as box and whisker plots (Min to Max, + represents the mean) from *N* = 4 independent experiments. ***P = 0.0001, two‐way ANOVA, Sidak's multiple comparisons test. (C) A2780‐Rab25 spheroids generated by the hanging drop method were embedded in 3 mg·mL^−1^ Geltrex, 3 mg·mL^−1^ collagen I and 25 μg·mL^−1^ fibronectin in the presence of DMSO, 5 or 10 μm ADAMTS5 inhibitor (TS5 i) and imaged live with a Nikon A1 confocal microscope up to day 2. Scale bar, 200 μm. Spheroid invasion area was quantified with imagej and normalised to DMSO day 1. Data are presented as box and whisker plots (Min to Max, + represents the mean) from *N* = 4 independent experiments. *P = 0.0207, ****P < 0.0001, two‐way ANOVA, Dunnett's multiple comparisons test. (D) A2780‐Rab25 cells were transfected with an siRNA targeting ADAMTS5 (si‐TS5) or a non‐targeting siRNA control (si‐nt), spheroids were generated, embedded, and imaged as in A. Scale bar, 200 μm. Spheroid invasion area was quantified with imagej and normalised to si‐nt day 1. Data are presented as box and whisker plots (Min to Max, + represents the mean) from *N* = 4 independent experiments. ***P = 0.0004, two‐way ANOVA, Sidak's multiple comparisons test. Image created with items adapted from Servier Medical Art, licensed under CC BY 4.0.

As ADAMTS5 was required for Rab25‐dependent cell migration, we investigated the role of ADAMTS5 in OC cell invasion in 3D systems. A2780‐DNA3 and A2780‐Rab25 cell spheroids were embedded into a matrix mix containing 3 mg·mL^−1^ Geltrex, 3 mg·mL^−1^ collagen I, and 25 μg·mL^−1^ fibronectin and allowed to invade for 2 days (Fig. 8A). The area of invading protrusions outside of the spheroid core was marked as the “invasion area” and was normalized to the core area at day 0. Consistent with previous reports [13, 26], the expression of Rab25 strongly promoted 3D invasion in A2780 cells (Fig. 8B). A2780‐Rab25 cell spheroids were then embedded into the matrix mix in the presence of DMSO, 5 or 10 μm ADAMTS5 active‐site inhibitor, and imaged at day 0, day 1, and day 2. Treatment with the ADAMTS5 inhibitor resulted in a dose‐dependent reduction in cell invasion (Fig. 8C). Consistently, ADAMTS5 knockdown significantly reduced A2780‐Rab25 cell 3D invasion, albeit to a smaller extent than the pharmacological inhibition (Fig. 8D).

**Fig. 9:**
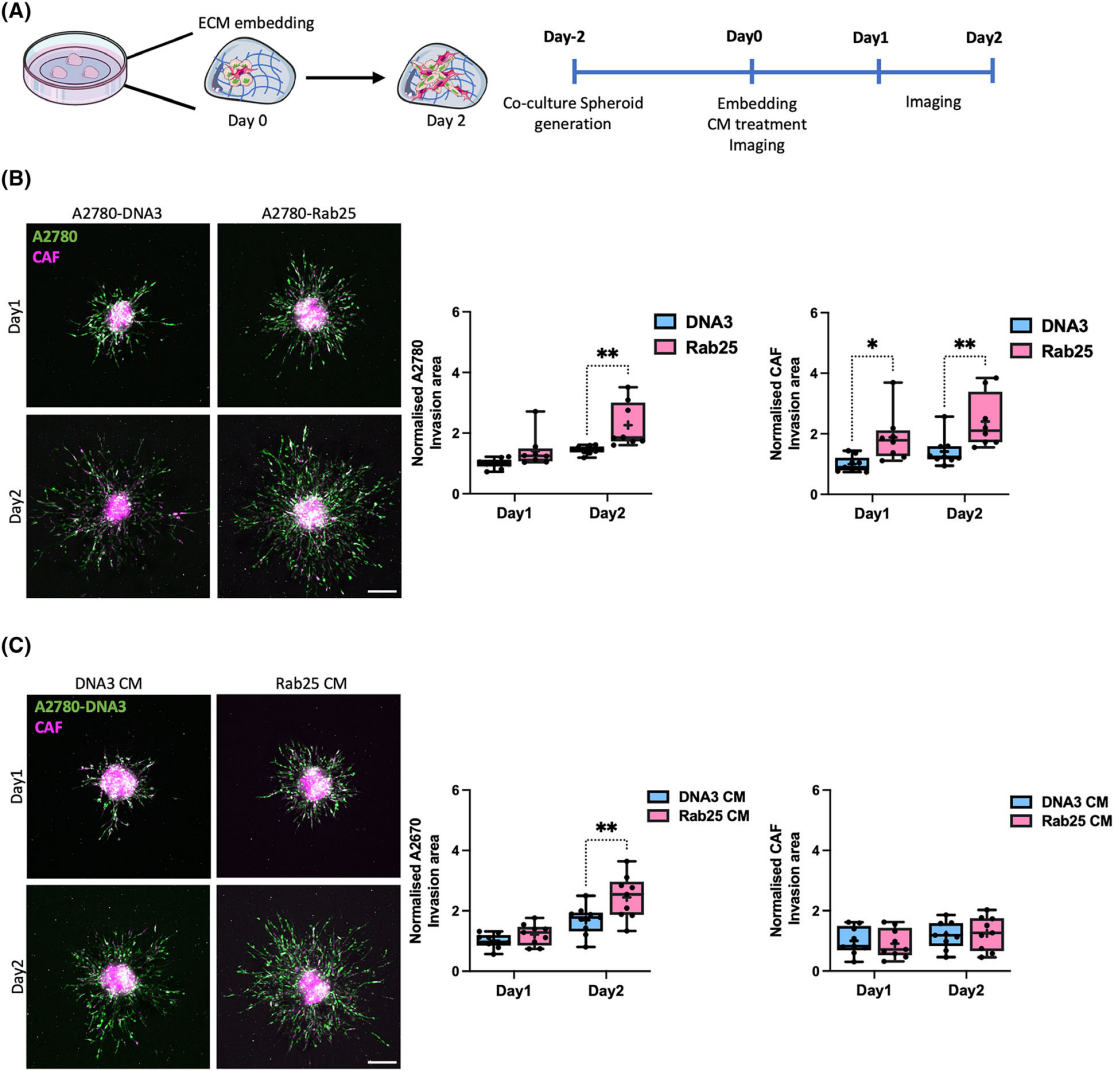
Rab25 expression promoted OC cell invasion in co‐culture with CAFs. (A) Schematic of 3D invasion assays. (B) A2780‐DNA3 and A2780‐Rab25 cells were labelled with Cell Tracker Green and CAFs were labelled with Cell Tracker Red. Co‐culture spheroids (1 : 1 ratio) generated by the hanging drop method were embedded in 3 mg·mL^−1^ Geltrex, 3 mg·mL^−1^ collagen I, and 25 μg·mL^−1^ fibronectin and imaged live with a Nikon A1 confocal microscope up to day 2. Scale bar, 200 μm. Spheroid invasion area was quantified with imagej and normalised to DNA3 day 1. Data are presented as box and whisker plots (Min to Max, + represents the mean) from *N* = 4 independent experiments. *P = 0.0173, **P < 0.008, two‐way ANOVA, Sidak's multiple comparisons test. (C) A2780‐DNA3/CAF co‐culture spheroids were generated and embedded as in B, treated with conditioned media (CM) generated by A2780‐DNA3 (DNA3) or A2780‐Rab25 (Rab25) cells and imaged live with a Nikon A1 confocal microscope up to day 2. Scale bar, 200 μm. Spheroid invasion area was quantified with imagej and normalised to DNA3 CM Day 1. Data are presented as box and whisker plots (Min to Max, + represents the mean) from *N* = 4 independent experiments. **P = 0.0044, two‐way ANOVA, Sidak's multiple comparisons test. Image created with items adapted from Servier Medical Art, licensed under CC BY 4.0.

**Fig. 10:**
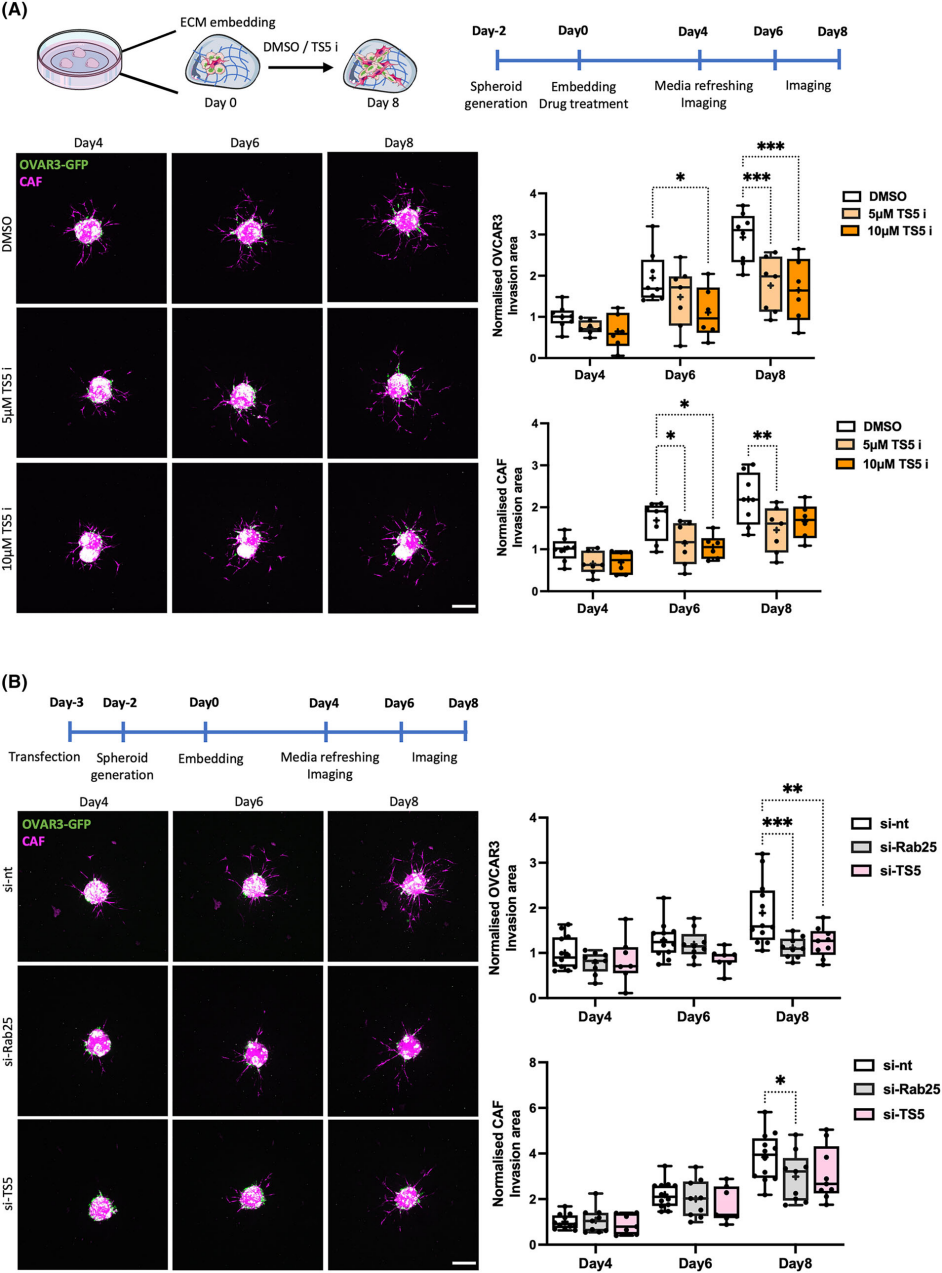
ADAMTS5 was required for OC cell invasion in co‐culture with CAFs. (A) OVCAR3 cells stably expressing nuclear GFP (H2B‐GFP) and cancer‐associated fibroblasts (CAFs) labelled with Cell tracker™ Red CMTPX (2 : 1 ratio) were generated, embedded in 3 mg·mL^−1^ Geltrex, 3 mg·mL^−1^ collagen I, and 25 μg·mL^−1^ fibronectin in the presence of DMSO, 5 or 10 μm ADAMTS5 inhibitor (TS5 i) and imaged live with a Nikon A1 confocal microscope up to day 8. Scale bar, 200 μm. Spheroid invasion area was quantified with imagej and normalised to DMSO day 4. Data are presented as box and whisker plots (Min to Max, + represents the mean) from *N* = 3 independent experiments. *P < 0.05, **P = 0.0035, ***P < 0.001, two‐way ANOVA, Dunnett's multiple comparisons test. (B) Co‐culture spheroids were generated with OVCAR3‐GFP cells (green) transfected with a non‐targeting control (si‐nt), Rab25‐targeting (si‐Rab25) or ADAMTS5 targeting (si‐TS5) si‐RNA and Cell tracker™ Red CMTPX (magenta) labelled CAFs (2 : 1 ratio), embedded in 3 mg·mL^−1^ Geltrex, 3 mg·mL^−1^ collagen I and 25 μg·mL^−1^ fibronectin and imaged live with a Nikon A1 confocal microscope up to day 8. Scale bar, 200 μm. The spheroid invasion area was calculated with imagej and normalised to si‐nt day 4. Data are presented as box and whisker plots (Min to Max, + represents the mean) from *N* = 4 independent experiments. *P = 0.0353, **P = 0.0012, ***P = 0.0001, two‐way ANOVA, Dunnett's multiple comparisons test. Image created with items adapted from Servier Medical Art, licensed under CC BY 4.0.

During OC metastasis, CAFs were found to form heterotypic spheroids with tumour cells and promote OC cell invasion through the secretion of proteases and ECM components [28, 29, 30]. To better investigate the role of CAFs in ADAMTS5‐mediated OC cell invasion, we established a co‐culture 3D spheroid model with Cell Tracker Red‐labelled CAFs and Cell Tracker Green‐labelled A2780 cells (Fig. 9A). As in the monoculture condition, the expression of Rab25 significantly increased A2780 cell invasion also in co‐culture with CAFs. Surprisingly, Rab25 over‐expression significantly promoted CAF invasion as well (Fig. 9B), suggesting that Rab25 might be involved in CAF/OC cell crosstalk. We then wanted to investigate whether Rab25 pro‐invasive function was driven by ADAMTS5 secretion. To do this, A2780‐DNA3 and CAF co‐culture spheroids were generated, embedded in the matrix mix, and allowed to invade for 2 days in the presence of conditioned media generated by either A2780‐DNA3 or A2780‐Rab25 cells. In agreement with our migration experiments, Rab25 conditioned media was sufficient to drive A2780 cell invasion. Interestingly, Rab25 conditioned media did not affect CAF invasion (Fig. 9C), indicating that CAF/OC cell crosstalk might require cell–cell interaction. Similarly, co‐culture spheroids were generated with Cell Tracker Red‐labelled CAFs and OVCAR3‐GFP cells, which do not invade out of the spheroids when grown as monoculture (data not shown). The spheroids were embedded into the matrix mix in the presence of DMSO, 5 or 10 μm of ADAMTS5 active‐site inhibitor, and imaged at day 4, day 6, and day 8. Consistent with the A2780‐Rab25 spheroid results, the invasion of OVCAR3 cells in co‐culture with CAFs was impaired by ADAMTS5 inhibition in a dose‐dependent manner, indicating that ADAMTS5 catalytic activity was required for Rab25‐overexpressing OC cell invasion. Interestingly, CAF invasion was also impaired by ADAMTS5 inhibition in co‐culture 3D spheroids (Fig. 10A).

In the TME, both tumour cells and stroma cells including CAFs were found to secrete proteases and remodel the ECM [6]. Therefore, it is possible that ADAMTS5 secreted from both OVCAR3 and CAFs promoted spheroid invasion. To investigate the contribution of ADAMTS5 secreted by cancer cells, OVCAR3‐GFP cells were transfected with a non‐targeting siRNA control, Rab25 targeting, or ADAMTS5 targeting siRNAs (Fig. S3B) and the 3D invasion of co‐culture spheroids was measured (Fig. 10B). While co‐culture spheroids generated from control siRNA transfected cells invaded into the surrounding matrix over time, both Rab25 and ADAMTS5 knockdown significantly reduced the invasion ability of OVCAR3 cells in co‐culture with CAFs (Fig. 10B). Importantly, the siRNA transfection resulted in a lasting reduction of Rab25 protein levels up to 11 days after transfection, corresponding to spheroid invasion day 8 (Fig. S3A). These data demonstrated that Rab25 and ADAMTS5 expression in cancer cells were required to promote spheroid invasion. Interestingly, Rab25 knockdown in OVCAR3 cells also reduced the invasion ability of CAFs in co‐culture, while ADAMTS5 knockdown resulted in a small but not statistically significant inhibition, indicating that Rab25 may be involved in mediating the crosstalk between OC cells and CAFs during metastasis. Altogether, these results show that ADAMTS5 was required for Rab25‐dependent invasion of OC cells in 3D systems.

**Fig. 11:**
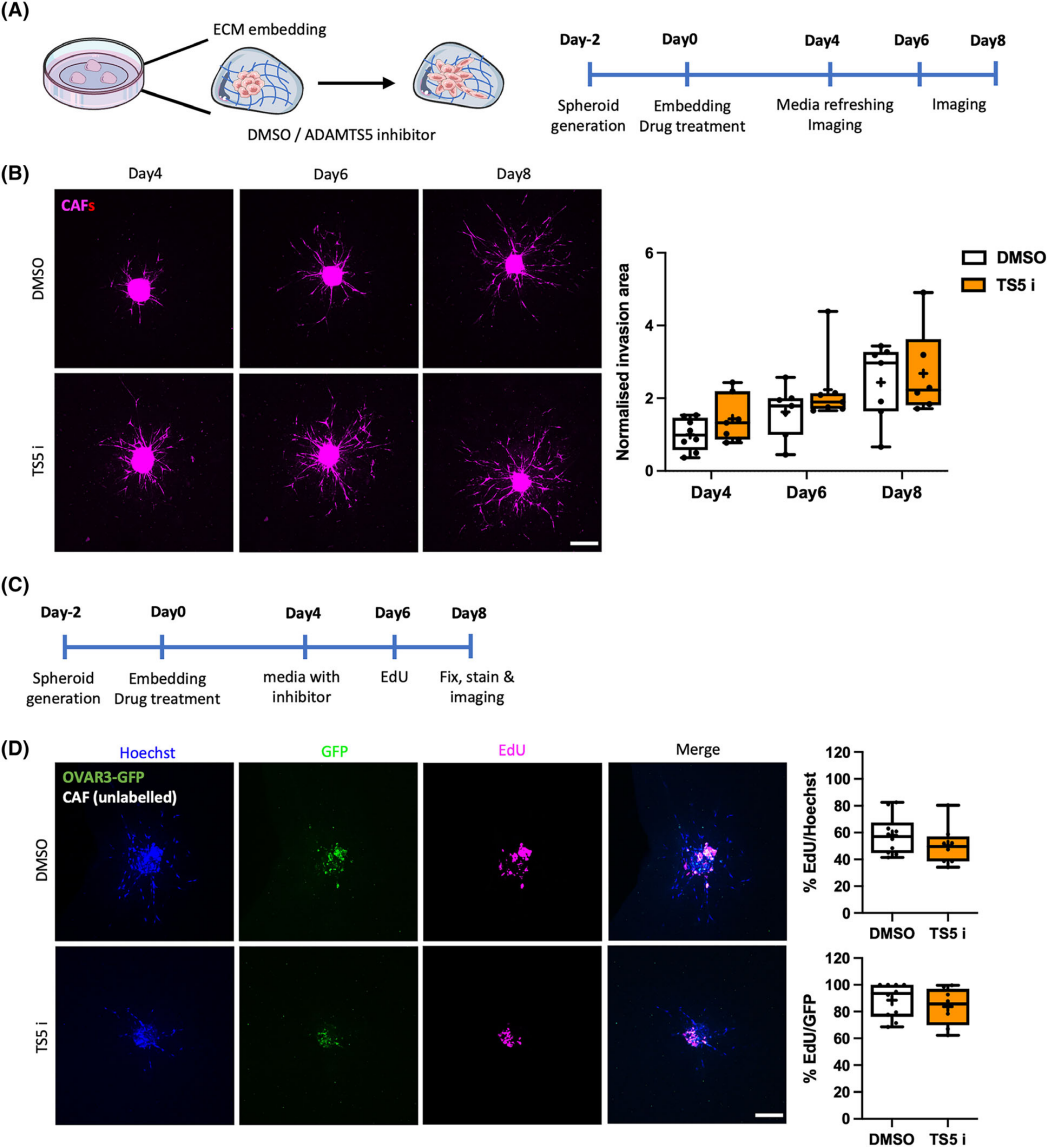
ADAMTS5 inhibition did not affect CAF spheroid 3D monoculture invasion nor cell proliferation in 3D. (A, C) Experimental timeline. (B) Spheroids generated with Cell tracker™ Red CMTPX labelled CAFs were embedded in 3 mg·mL^−1^ Geltrex, 3 mg·mL^−1^ collagen I, and 25 μg·mL^−1^ fibronectin in the presence of DMSO control or 10 μm ADAMTS5 inhibitor (TS5 i) and imaged live with a Nikon A1 confocal microscope up to day 8. Scale bar, 200 μm. Spheroid invasion area was quantified with imagej and normalized to DMSO day 4. Data are presented as box and whisker plots (Min to Max, + represents the mean) from *N* = 3 independent experiments. (D) Spheroids generated with OVCAR3‐GFP cells (green) and non‐labelled CAFs (2 : 1 ratio) were embedded in 3 mg·mL^−1^ Geltrex, 3 mg·mL^−1^ collagen I, and 25 μg·mL^−1^ fibronectin and cultured in the presence of DMSO or 10 μm ADAMTS5 inhibitor (TS5 i). EdU was added at day 6, spheroids were fixed on day 8, stained with EdU detection reagent (magenta) and Hoechst 33342 (blue) and imaged with a Nikon A1 confocal microscope. Scale bar, 200 μm. The percentage of EdU positive cells was calculated against Hoechst or GFP. Data are presented as box and whisker plots (Min to Max, + represents the mean) from *N* = 3 independent experiments.

In the OVCAR3/CAF co‐culture model, CAFs were observed at the tips of invading strands, which seemed to lead to the invasion of OVCAR3 cells. Since the inhibition of ADAMTS5 reduced the invasion capacity of both CAFs and OVCAR3 cells, it is possible that the zinc‐chelating ADAMTS5 inhibitor indirectly suppressed the invasion of OVCAR3 cells by preventing CAF invasion. To investigate this, monoculture CAF spheroids were generated and embedded in the presence of DMSO or 10 μm active‐site ADAMTS5 inhibitor (Fig. 11A). Interestingly, CAF invasion was not affected by ADAMTS5 inhibition in monoculture (Fig. 11B), suggesting that ADAMTS5 might control CAF/OVCAR3 cell crosstalk. Given the timeframe of the 3D spheroid assays, changes in cell proliferation could affect the quantification of the invasion area. To investigate whether ADATMS5 inhibition perturbed cell growth, we performed a 5‐ethynyl‐2′‐deoxyuridine (EdU) incorporation assay in OVCAR3/CAF spheroids (Fig. 11C). EdU is a thymidine analogue, which is incorporated in the DNA during replication in the S phase of the cell cycle. As a result, only OVCAR3 cells were positive for EdU, and the percentage of EdU positive cells was not affected by ADAMTS5 inhibition (Fig. 11D), indicating that ADAMTS5 was required for 3D invasion without affecting cell proliferation.

### ADAMTS5 is a poor prognosis factor for OC

**Fig. 12:**
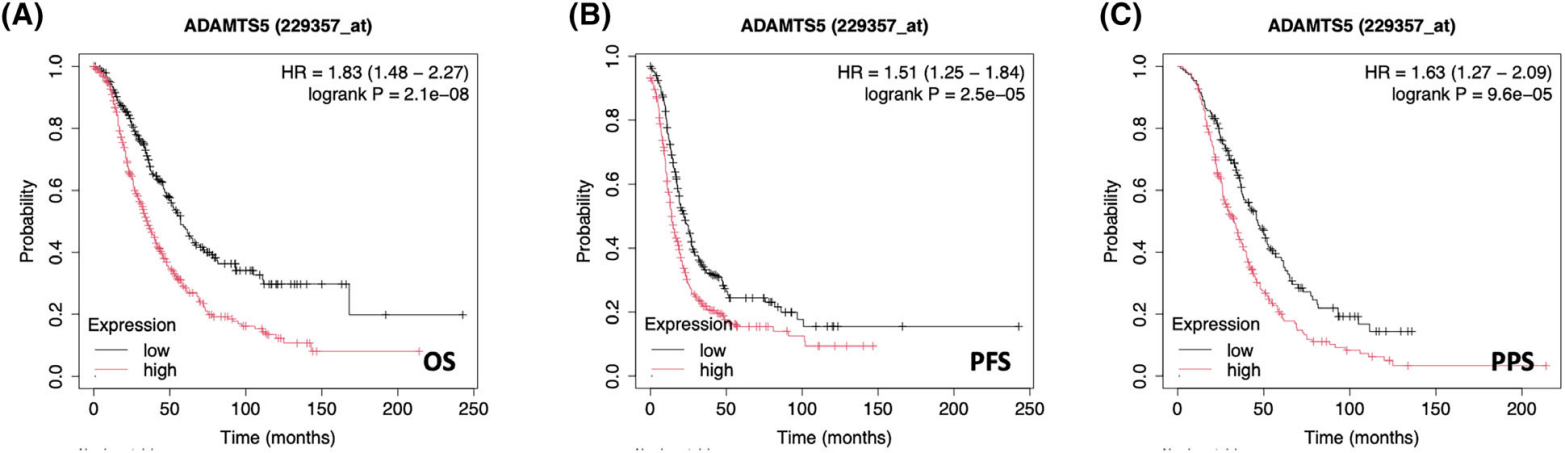
ADAMTS5 expression correlated with poor prognosis of OC patients. (A–C) 655 ovarian cancer patients were stratified into low and high ADAMTS5 expression. The Kaplan–Meier analysis compared (A) overall survival (OS), (B) progression‐free survival (PFS) and (C) palliative performance scale (PPS) of OC patients with tumours expressing high ADAMTS5 levels (red) with those expressing low ADAMTS5 levels (black).

Given the role of ADAMTS5 in the migration and invasion of OC cells, we looked at the relationship between ADAMTS5 expression and survival outcomes of OC patients. Interestingly, high ADAMTS5 expression correlated with reduced overall survival (OS), progression‐free survival (PFS) and the palliative performance scale (PPS) (Fig. 12A–C). Taken together, these data suggest that ADAMTS5 could promote metastasis by enhancing OC cell migration and invasion, leading to poor prognosis outcomes for OC patients. Therefore, ADAMTS5 might represent a novel therapeutic target to prevent OC metastasis.

## Discussion

**Fig. 13:**
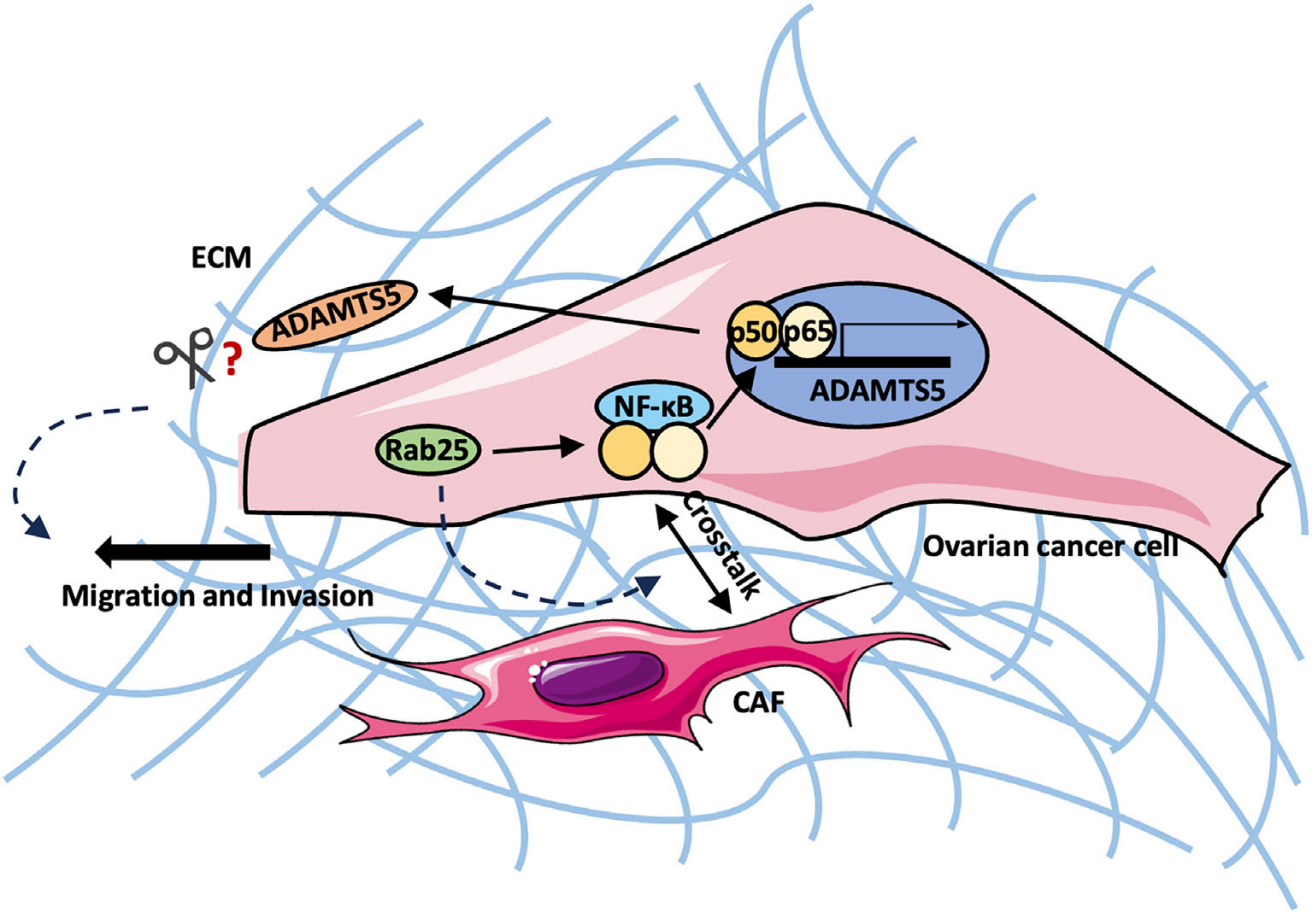
Working model. Upregulated Rab25 induced ADAMTS5 expression through an NF‐κB‐dependent signalling pathway in OC cells. Secreted ADAMTS5 promoted OC cell migration and invasion in a proteolytic activity‐related manner. Rab25 could also be involved in controlling the crosstalk between OC cells and CAFs in the TME. Image created with items adapted from Servier Medical Art, licensed under CC BY 4.0.

Altered ECM composition and structure have been widely detected in the TME of multiple solid tumours, including OC [29]. These changes are mainly caused by the dysregulation of ECM‐modifying enzymes, which can be secreted by both tumour cells and CAFs [6, 31]. Here, we demonstrated that Rab25 induced ADAMTS5 expression in OC cells by upregulating the activity of NF‐κB in an AKT‐ and ERK‐independent manner. Remarkably, we showed that ADAMTS5 pharmacological inhibition or knockdown significantly reduced OC cell migration and invasion without affecting cell proliferation (Fig. 13).

We found that both endogenous and exogenous overexpression of Rab25 increased ADAMTS5 expression, while the presence of the ECM further increased ADAMTS5 levels in A2780 but not OVCAR3 cells. This could be due to the different origins of the cells, as the A2780 cell line was established from an ovarian endometrioid adenocarcinoma sample, while OVCAR3 cells were derived from the ascitic fluid of an OC patient with high‐grade ovarian cancer [32]. Unfortunately, after performing the conditioned media experiments in A2780 cells with a validated batch of the anti‐ADAMTS5 antibody, the new batch did not work; therefore, we were unable to test ADAMTS5 secretion in the conditioned media of OVCAR3 cells.

In agreement with data in chondrogenic cells, here we showed that NF‐κB was required for ADAMTS5 expression in OC cells overexpressing Rab25. Indeed, both Rab25 downregulation and NF‐κB inhibition resulted in a ~ 40–50% reduction in ADAMTS5 mRNA levels, indicating that additional pathways are likely to be involved in controlling the expression of ADAMTS5. While a direct interaction between NF‐κB and the ADAMTS5 promoter has been reported [18], we cannot rule out the possibility of an indirect regulation of ADAMTS5 expression, via the modulation of cytokines, such as IL‐1, IL‐6, and IL‐8 downstream of NF‐κB activation [33]. Indeed, IL‐1β and IL‐6 were shown to induce ADAMTS5 expression in osteoarthritis [34]. Although NF‐κB has been shown to be regulated by PI3K/AKT and MAPK/ERK pathways in OC cells [24, 35], none of these were dependent on Rab25 in our settings. This indicates that Rab25 might activate NF‐κB via other signaling pathways, which warrants further investigation. It has been suggested that the mammalian target of rapamycin complex 1 (mTORC1) might regulate NF‐κB. Interestingly, we have demonstrated that Rab25 promotes mTORC1 activation in OC cells [26], suggesting that Rab25 might control NF‐κB in a mTORC1‐dependent manner.

We found that the catalytic activity of ADAMTS5 was required for cell migration and invasion in the presence of ECM, as we could inhibit both processes using pharmacological inhibitors that blocks ADAMTS5's ability to cleave its substrates. While both knockdown and pharmacological inhibitor treatment resulted in a comparable inhibition of cell migration on CDM, the ADAMTS5 zinc‐chelating inhibitor had a stronger effect on reducing 3D invasion compared to the siRNA transfection (43% compared to 32% reduction at day 6). This could be due to the fact that zinc‐chelating ADAMTS inhibitors are not entirely selective, as they generally also inhibit MMP activity [36], which might play a more prominent role in 3D invasion. Interestingly, a newly developed and highly selective non‐zinc chelating ADAMTS5 inhibitor (4b) reduced pseudopod elongation and directed cell migration, supporting the notion that ADAMTS5's proteolytic activity is required to drive OC cell migration. ADAMTS5 was found to cleave aggrecan, brevican, neurocan, and versican [37]. Among them, versican was found to be increased in OC stroma, which was shown to stimulate OC cell migration and invasion and promote peritoneal metastasis [9, 38, 39]. In addition, versican secreted by primary ovarian CAFs was found to promote OC cell invasion [40]. Since blocking the ADAMTS cleavage site on versican reduced the migration of glioma cancer cells [41], it is possible that ADAMTS5 could also drive OC cell migration by cleaving its substrate versican. Interestingly, versican cleavage by ADAMTSs results in the release of a matrikine fragment, versikine, which has been shown to promote glioma cell migration [41]. Further work will elucidate the contribution of versican cleavage to OC cell migration.

Interestingly, we found that NF‐κB was required for Rab25‐dependent cell migration on CDM. This is consistent with previous findings, highlighting a pro‐migratory role of NF‐κB in lung, breast, and ovarian cancer cells [42, 43, 44, 45]. In addition, NF‐κB inhibition also significantly impaired metastasis formation in mouse xenograft models [43]. The fact that we were able to rescue the migration of NF‐κB‐inhibited OC cells by adding ADAMTS5‐containing conditioned media suggests that NF‐κB pro‐migratory activities are at least in part mediated by the upregulation of ADAMTS5 expression.

Additionally, our data showed that the knockdown of Rab25, and to a lesser extent ADAMTS5, in OC cells reduced CAF invasion in co‐culture spheroids, while ADAMTS5 inhibition did not affect CAF invasion in mono‐culture spheroids, suggesting a potential role of Rab25 and ADAMTS5 in controlling cancer cell/CAF crosstalk. To note, Rab25 is epithelial‐specific and, therefore, not expressed in CAFs [46]. Transforming growth factor β (TGFβ) has a key role in sustaining CAF phenotypes. Interestingly, members of the ADAMTS family, including ADAMTS1, 6, and 10, were found to control TGFβ signalling [47, 48]. Moreover, TFGβ1 expression has been reported to be upregulated in Rab25‐expressing cells [10], suggesting that ADAMTS5 and Rab25 might control OC cell/CAF crosstalk via TGFβ signalling.

ADAMTS5 has been reported to either promote or inhibit tumour formation in a context‐dependent manner. Consistent with our analysis showing a correlation between high ADAMTS5 expression and poor patient prognosis in OC, ADAMTS5 was shown to be a tumour promoter in glioblastoma [49], non‐small cell lung cancer [50] and head and neck cancer [51], while in melanoma and gastric carcinoma, ADAMTS5 was found to suppress tumour progression by inhibiting angiogenesis in a catalytic activity‐independent manner [52, 53].

In conclusion, our study demonstrated that Rab25 induced ADAMTS5 expression in an NF‐κB‐dependent manner in OC cells. ADAMTS5 was required for OC cell migration and invasion, and its expression correlated with reduced OC patient survival, suggesting that it might be a novel regulator of OC metastasis. Since small molecule inhibitors of ADAMTS5 are in phase III clinical trials for osteoarthritis treatment [36], our data suggest that these could be repurposed to prevent OC progression.

## Materials and methods

### Reagents

Primary antibodies for western blotting: ADAMTS5 (ab41037; Abcam, Cambridge, UK), Rab25 (18139‐1‐AP; Proteintech, Manchester, UK), AKT (#4691; Cell Signalling Technology, Leiden, the Netherlands), p‐AKT (#4060; Cell Signalling Technology), ERK1/2 (#9102; Cell Signalling Technology), p‐ERK (#9101; Cell Signalling Technology), NF‐κB (10745‐1‐AP; Proteintech), GAPDH (SC‐47724; Santa Cruz Biotechnology, Dallas, Texas, USA) and α‐Tubulin (T9026; Sigma‐Aldrich, Merck Life Science UK, Gillingham, Dorset, UK). Secondary antibodies for western blotting: IR Dye 680 LT goat anti‐Rabbit IgG antibody (926‐68021; LICOR Biotechnology, Lincoln, Nebraska, USA), IR Dye 800 CW goat anti‐Mouse antibody (926‐32210; LICOR Biotechnology). Primary and secondary antibodies for immunofluorescence: NF‐κB/p65 (10745‐1‐AP; Proteintech), Alexa Fluor® 488 goat anti‐rabbit IgG (H + L) (A11034; Invitrogen, Thermo Fisher Scientific, Waltham, MA, USA), Alexa Fluor® 555 donkey anti‐rabbit IgG (H + L) (A31572; Life Technologies). Primers for RT‐qPCR (Qiagen, Manchester, UK): ADAMTS5 (Hs_ADAMTS5_1_SG), Rab25 (Hs_RAB25_1_SG), GAPDH (Hs_GAPDH_1_SG), CXCL8 (Hs_CXCL8_1_SG). ON‐TARGETplus SMARTpool siRNAs (Dharmacon, Horizon Discovery, Cambridge, UK): ADAMTS5 (L‐005775‐00‐0005), Rab25 (L‐010366‐00‐0005), siGENOME non‐targeting control siRNA #4. The ADAMTS5 inhibitor (5‐[[5‐[[(4‐Chlorophenyl)methyl]thio]‐1‐methyl‐3‐(trifluoromethyl)‐1H‐pyrazol‐4‐yl]methylene]‐2‐thioxo‐4‐thiazolidinone;(E)‐5‐((5‐((4‐Chlorobenzyl)thio)‐1‐methyl‐3‐(trifluoromethyl)‐1H‐pyrazol‐4 yl)methylene)‐2‐thioxothiazolidin‐4‐one CAS no.: 929634‐33‐3) was from MedChemExpress, MedChemTronica, Sollentuna, Sweden reported to be a potent ADAMTS‐5 inhibitor, with an IC50 of 1.1 μm and > 40‐fold functional selectivity over ADAMTS4 [54, 55]. 4b was synthesised as before [27]. 4b is a non zinc‐chelating inhibitor directed against residues K532 and K533 in the ADAMTS5 disintegrin‐like domain. 4b inhibits versicanase activity of ADAMTS5 with an IC50 of 9.4 μm while sparing ADAMTS4 [27]. TNFα was from Thermo Fisher Scientific.

### Cell culture

The OC cell lines A2780 (RRID: CVCL_0134), OVCAR3 (RRID: CVCL_0465), OVCAR4 (RRID: CVCL_1627) and omental cancer‐associated fibroblast (CAFs) were cultured in RPMI‐1640 medium (Gibco, Thermo Fisher Scientific) supplemented with 10% (v/v) foetal bovine serum (FBS; Gibco, Thermo Fisher Scientific) and 1% (v/v) penicillin/streptomycin (Pen/Strep, Gibco; Thermo Fisher Scientific). SKOV3 (RRID: CVCL_0532) and telomerase‐immortalised human dermal fibroblasts (TIFs) were cultured in high glucose Dulbecco's modified Eagle medium (DMEM; Gibco, Thermo Fisher Scientific) supplemented with 10% (v/v) FBS and 1% (v/v) Pen/Strep. A2780‐DNA3 and Rab25 cells generated in G. Mill's lab, as described in Ref. [12] were a gift from J. Norman's lab (Cancer Research UK Scotland Institute). To maintain the overexpression of Rab25, cell lines were selected with 0.4 mg·mL^−1^ G418 for a week every 10 passages. OVCAR3 cells were a gift from P. Caswell's lab (The University of Manchester) and were STR profiled. The hTERT immortalised omental CAFs from S. Zanivan's lab (Cancer Research UK Scotland Institute, Glasgow) were generated as described in Ref. [16]. OVCAR4 was a gift from S. Zanivan, and SKOV3 was from ATCC, Gaithersburg, Maryland, USA. All cell lines were maintained at 37 °C in 5% CO_2_, passaged every 3–4 days, and routinely tested for mycoplasma contamination. A2780 and OVCAR3 were STR profiled.

### Cell‐derived matrix generation

Cell‐derived matrix generation has been previously described [56]. Briefly, plates were coated with 0.2% (v/v) gelatin (Sigma‐Aldrich) in PBS for 1 h at 37 °C, followed by crosslinking with 1% (v/v) sterile glutaraldehyde (Sigma‐Aldrich) in PBS for 30 min at room temperature (RT). The glutaraldehyde was quenched with 1 m sterile glycine (Sigma‐Aldrich) in dH_2_O for 20 min at RT, and complete medium was used for equilibration for 30 min at 37 °C. TIFs and CAFs were then seeded on top of the coated plates (5 x 10^5^ cells/well in 6 well plates and 2.5 x 10^5^ cells/well in 12 well plates). After reaching confluency, the media was replaced with complete media containing 50 μg·mL^−1^ ascorbic acid (Sigma‐Aldrich) and was refreshed every other day. TIFs were kept in DMEM complete media with ascorbic acid for 9 days to secrete CDM. To extract TIF‐CDM, triton extraction buffer (20 mm NH_4_OH and 0.5% (v/v) Triton X‐100 in PBS containing Ca^2+^ and Mg^2+^ (PBS^++^)) was added on top and left at RT until all cells were removed. Then, 10 μg·mL^−1^ DNase I in PBS^++^ was added, and the plates were incubated at 37 °C for 1 h. CAFs were kept in Human Plasma‐Like Medium (HPLM; Gibco, Thermo Fisher) with ascorbic acid for 11 days. The CDM was extracted with PLA2 extraction buffer (50 mm Tris–HCl pH 8, 150 mm NaCl, 1 mm MgCl_2_, 1 mm CaCl_2_, 0.5% sodium deoxycholate and 20 unit·mL^−1^ PLA2) at 37 °C for 1 h. Then, 10 μg·mL^−1^ DNase I in PBS^++^ was added, and the plates were incubated at 37 °C overnight. The CDMs were stored at 4 °C in PBS^++^ and were used within 2 weeks.

### Conditioned media harvest

A2780 cells were seeded on plastic and incubated at 37 °C in 5% CO_2_ for 3 days. Then, the conditioned media (CM) was centrifuged at 300 ***g*** for 10 min, 2000 ***g*** for 10 min, and 10 000 ***g*** for 30 min at 4 °C. The supernatant was transferred into new falcon tubes and stored at 4 °C for up to a week.

### Western blotting

Cells seeded on plastic were lysed in SDS‐lysis buffer (50 mm Tris PH7, 1% SDS in dH_2_O) and cells seeded on CDM were lysed with triton extraction buffer (20 mm NH_4_OH and 0.5% (v/v) Triton X‐100 in PBS containing Ca^2+^ and Mg^2+^ (PBS^++^)). Cell lysates were then centrifuged down through QiaShredder columns (Qiagen) at full speed for 5 min. To analyse CM proteins, A2780‐DNA3 and Rab25 cells were seeded in complete media and cultured at 37 °C in 5% CO_2_, and the culture media was changed into serum‐free media ~ 4 h after seeding. After 3 days, the CMs were subjected to the 3‐step centrifugation protocol as described above. The CMs were further concentrated using Amicon Ultra® – 4 Centrifugal filters (3000 or 10 000 MWCO PES; Merck, Millipore, Watford, Hertfordshire, UK). For both cell lysate and concentrated CM, the samples were mixed with 4× NuPAGE buffer containing 1 mm DTT and boiled at 70 °C for 5 min. Twenty‐five microliter of the samples and 1 μL of the protein ladder (New England BioLabs, Ipswich, MA, USA) were loaded into a Bio‐Rad 4–15% Mini‐PROTEAN precast polyacrylamide gel (Bio‐Rad Laboratories, Hercules, CA, USA), then run at 100 V for 75 min in 1× running buffer (25 mm Tris, 192 mm glycine and 1% SDS in dH_2_O). The membrane transfer was performed in Towbin transfer buffer (25 mm Tris, 192 mm glycine and 20% methanol (v/v) in dH_2_O, pH 8.3) at 100 V for 75 min. The membranes were then blocked with 5% (w/v) milk in 1× TBST (50 mm Tris HCl, 150 mm NaCl and 0.5% (w/v) Tween 20 in dH_2_O) for 1 h at RT. The membranes were incubated overnight with primary antibodies (ADAMTS5 1 : 250, Rab25 1 : 600, AKT 1 : 1000, p‐AKT 1 : 1000, ERK 1 : 1000, p‐ERK 1 : 1000) together with GAPDH (1 : 1000) in 1× TBST at 4 °C, alternatively the membranes were incubated with the anti‐NF‐κB primary antibody (1 : 3000) at RT for 1.5 h, followed by a 1‐h incubation at RT with secondary anti‐mouse IgG LICOR IR Dye 800 (1 : 30 000) and anti‐rabbit IgG LICOR IR Dye 680 (1 : 20 000) in TBST with 0.01% (w/v) SDS. The membranes were then imaged with a LICOR Odyssey Sa system, and the intensity of the protein bands was quantified with image studio lite software (LICOR Biotechnology, Lincoln, Nebraska USA).

### RT‐qPCR

mRNA was extracted from snap‐frozen cell pellets according to the manufacturer's protocol (RNeasy^®^ Mini – Qiagen). cDNA was then synthesised using the High‐Capacity cDNA Reverse Transcription Kit (Fisher) following the manufacturer's protocol. Then, a final concentration of 1× QuantiNova SYBR^®^ Green PCR Kit (Qiagen) master mix and 1× QuantiTect^®^ Primer Assay for target genes (ADAMTS5, Rab25 and GAPDH) was prepped in RNase‐free water. Seven microliter of loading master mix and 3 μL of cDNA solution (1 : 100 dilution, 5 ng·μL^−1^ final conc.) were loaded into a 384‐well plate. The RT and blank water controls were tested for all target genes in each individual run. Quantstudio 12 K flex real‐time PCR system was used to analyze the samples in the SYBR^®^ mode. Expression levels of the target genes were calculated using 2^−∆∆Ct^ method (2^−(∆Ct Target gene − ∆Ct Housekeeping gene)^) with GAPDH as the housekeeping gene. Each sample was tested in three technical replicates.

### siRNA transfection

Two microliter Dharmafect I (Dharmacon) was mixed with 198 μL FBS‐ and antibiotic‐free RPMI medium and incubated for 5 min at RT. Meanwhile, 2.5 μL of 20 μm siRNA and 197.5 μL FBS‐ and antibiotic‐free RPMI medium were mixed, added to the Dharmafect solution, and incubated for 20 min at RT. The 400 μL solution was then added on top of the cells and topped up to 2 mL complete medium without Pen/Strep. Twenty‐four hours after transfection, the media were changed to complete medium without Pen/Strep. siGENOME non‐targeting control siRNA #4 was used as a non‐targeting control.

### Immunofluorescence

Thirty‐five millimeter glass‐bottom dishes (12 mm glass diameter; Wuxi NEST Biotechnology, Jiangsu, China) were coated with 0.1 mg·mL^−1^ Geltrex (Gibco) to facilitate cell adhesion. Cells were fixed with 4% (v/v) paraformaldehyde (PFA; Thermo Fisher Scientific) in PBS for 15 min and permeabilised with 0.25% (v/v) Triton X‐100 in PBS for 5 min at RT. Cells were blocked with 1% (w/v) BSA in PBS for 1 h, stained with primary anti‐NF‐κB/p65 antibody (1 : 100 in 1% BSA/PBS) for 1 h at RT, and incubated with secondary antibody (Alexa Fluor® 488 goat anti‐rabbit IgG (H + L) or Alexa Fluor® 555 donkey anti‐rabbit IgG (H + L), 1 : 1000 in 1% BSA/PBS) for 45 min. Since GFP‐OVCAR3 cells were used in these experiments, the Alexa Fluor 488‐conjugated secondary antibody was used for A2780 cells, while the Alexa Fluor 555‐conjugated one was used for OVCAR3 cells. For simplicity, NF‐κB/p65 staining is presented in green for both cell lines in the results section. The actin cytoskeleton was stained with Phalloidin Alexa Fluor 555 (1 : 400 in PBS) or Alexa Fluor 647 (1 : 300 in PBS) for 10 min. Finally, Vectashield mounting agent containing DAPI was added on top. Images were taken with a Nikon A1 confocal microscope, CFI Plan Apochromat VX 60× oil immersion objective, and the integrated density was measured in fiji/imagej [57].

### Cell migration assay

0.5 × 10^5^ cells/well were seeded on plastic or 12‐well plates containing CDM and allowed to spread for 4 h before imaging. For the CM migration assays, the cells were seeded in complete media mixed with CM (1 : 1 dilution). Where indicated, DMSO or inhibitors were added to the culture media before imaging. Cells were imaged live with a Nikon widefield live‐cell system (Nikon Ti eclipse with Oko‐lab environmental control chamber) with a Plan Apo 10× objective (NA 0.75). Images were acquired every 10 min for 16 h. For each condition, 5 positions were randomly imaged. Cell migration was manually tracked with the imagej plugin Manual Tracking [57, 58], and the migration velocity and directionality were calculated with the chemotaxis tool plugin (https://ibidi.com/chemotaxis‐analysis/171‐chemotaxis‐and‐migration‐tool.html). Pseudopod length, which describes the distance between nuclei and the tip of the pseudopod in the direction of cell migration, was measured with the “Straight” tool in imagej.

### 3D spheroid invasion assay

Spheroids were generated using the hanging drop method as previously described [59]. Briefly, A2780‐DNA3, A2780‐Rab25 cells, and CAFs were labelled with either Cell tracker™ Red CMTPX (Thermo Fisher Scientific, 1 : 3000 in FBS‐free media) or Cell tracker™ Green CMFDA (Thermo Fisher Scientific, 1 : 6000 in FBS‐free media) at 37 °C for 1 h. OVCAR3 cells were transfected with the pCAG‐H2B‐GFP plasmid (#184777; Addgene, Watertown, MA, USA) using Invitrogen™ Lipofectamine™ 2000 Transfection Reagent (Thermo Fisher Scientific) following the manufacturer's protocol, and a stable cell line was established. Then, 1 × 10^5^ (monoculture), 1 × 10^5^ (co‐culture, A2780‐Rab25:CAF = 1 : 1) or 2.1 × 10^5^ (co‐culture, OVCAR3:CAF = 2 : 1) labelled cells were suspended in 2 mL of medium containing 20 μg·mL^−1^ of soluble collagen I (Viscofan BioEngineering, Weinheim, Germany) and 4.8 mg·mL^−1^ of Methyl Cellulose (MTC; Sigma‐Aldrich). Twenty microliter drops of the cell suspension were then hung on the lid of a 10 cm tissue culture dish to generate the spheroids. After 48 h at 37 °C, the spheroids were harvested, embedded into 45 μL of a matrix mix containing 3 mg·mL^−1^ collagen I (Ibidi GmbH, Gräfelfing, Germany), 3 mg·mL^−1^ Geltrex (Gibco) and 25 μg·mL^−1^ Fibronectin (Sigma‐Aldrich), and placed on a 35 mm glass‐bottom dish. During matrix polymerisation, the dishes were incubated at 37 °C upright for 2 min, then flipped upside down and incubated for another 2 min. After 5 up and down flips (10 min in total), the dishes were kept upside down and incubated at 37 °C for 20–25 min until the matrices polymerised. For pharmacological inhibition, spheroids were cultured in complete media containing DMSO, 5 or 10 μm ADAMTS5 zinc‐chelating inhibitor. For CM treatment, spheroids were cultured in complete media mixed with CM (1 : 1 dilution). Spheroids were imaged live with a Nikon A1 confocal microscope, CFI Plan Fluor 10× objective (NA 0.3). The fluorescent images were thresholded in imagej, and the areas of the spheroid cores and the total area were measured. The invasion area was then calculated by total area – core area, and the invasion area was normalised to the core area. Data are presented as normalised to day 1 (for imaging up to day 2) or day 4 (for imaging up to day 8), meaning that the quantification reflects the increase in invasion between day 1 and day 2 or day 4 and day 8.

### 3D EdU incorporation assay

Co‐culture spheroids were generated with OVCAR3‐GFP cells and unlabelled CAFs, embedded and treated with DMSO or ADAMTS5 inhibitor as described above. After 6 days, a final concentration of 10 μm EdU solution was added to the media. After 2 days, the spheroids were fixed with 4% PFA containing Hoechst 33342 (Thermo Fisher Scientific, 1 : 500) at 37 °C for 20 min. Then, the spheroids were permeabilised with IF wash buffer (46 mm NaN_3_, 0.1% (w/v) BSA, 0.2% (v/v) Triton‐X 100 and 0.04% (v/v) Tween‐20 in PBS) for 2 h at RT. The spheroids were then stained with Click‐iT EdU Imaging Kits (Invitrogen) at 4 °C overnight. The spheroids were washed twice with PBS and imaged with a Nikon A1 confocal microscope, CFI Plan Fluor 10× objective (NA 0.3) and the thresholded area of Hoechst, GFP, and EdU signal were measured in imagej.

### Survival analysis

The survival analysis was performed with Kaplan–Meier plotter (https://kmplot.com/analysis/) [60], using ovarian cancer mRNA gene‐chip data. Sources for the databases include Gene Expression Omnibus (GEO), European Genome‐Phenome Archive (EGA), and The Cancer Genome Atlas (TCGA).

### Statistical analysis

Graphs were generated with graphpad prism software, Boston, MA, USA (version 9.1.0). Bar graphs are presented as super‐plots [61], where single data points from individual experiments are presented in different shades of blue or orange and the means of each biological replicate are shown as black dots. Three technical replicates were performed for each biological replicate. To compare two datasets, the Mann–Whitney test was used; to compare more than two datasets, one‐way ANOVA (Kruskal–Wallis test) was used when there was one independent variable, while 2‐way ANOVA (Dunnett's test) was performed when there were two independent variables.

## Conflict of interest

The authors declare no conflict of interest.

## Authors contributions

SY and ER contributed to conceptualisation; SY, RB, and ER contributed to data curation; SY, RB, and JA contributed to formal analysis; ER contributed to funding acquisition; SY, RB, and JA contributed to investigation; SY, RB, JA, DC, AR, EN, and SS contributed to methodology; ER contributed to project administration; ER, EN, and AR contributed to supervision; SY, ER, SS, and EN contributed to writing, review and editing.

## Peer review

The peer review history for this article is available at https://www.webofscience.com/api/gateway/wos/peer‐review/10.1111/febs.70080.

## Supporting information


**Fig. S1.** Rab25 did not affect LRP1 expression in OC cells.
**Fig. S2.** Rab25 protein expression in OC cells.
**Fig. S3.** Rab25 and ADAMTS5 KD efficiency in OVCAR3 cells.

## Data Availability

The data that supports the findings of this study are available in Figs 1, 2, 3, 4, 5, 6, 7, 8, 9, 10, 11, 12 and the Supporting Information of this article (Figs S1–S3).
