# Supplementary material for: The protease ADAMTS5 controls ovarian cancer cell invasion, downstream of Rab25
Source: FEBS J. 2025 Mar 31;292(17):4491–515. doi: 10.1111/febs.70080 (PMC12414872; doi:10.1111/febs.70080)
Supplement: Supplementary file 1 — Fig. S1. Rab25 did not affect LRP1 expression in OC cells. Fig. S2. Rab25 protein expression in OC cells. Fig. S3. Rab25 and ADAMTS5 KD efficiency in OVCAR3 cells. [file FEBS-292-4491-s001.pdf]

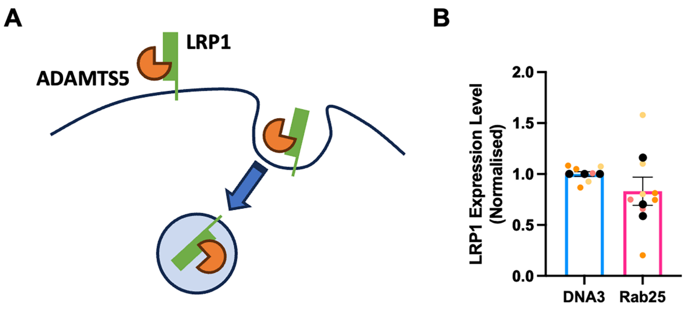

Figure S1. Rab25 did not affect LRP1 expression in OC cells. (A) Schematic, role of LRP1 in ADAMTS5 endocytosis. **(B)** LRP1 mRNA levels were measure by qPCR in A2780-Rab25 cells. The data were normalised to A2780-DNA3 cells. Data are mean ± SEM from N=3 independent experiments. The black dots represent the mean of individual experiments.

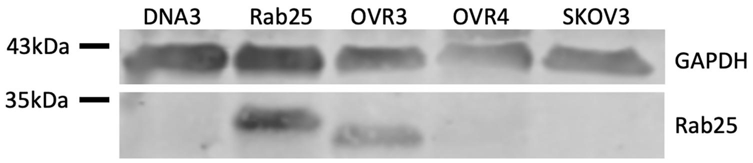

Figure S2. Rab25 protein expression in OC cells. A2780-DNA3, A2780-Rab25, OVCAR3, OVCAR4 and SKOV3 cells were seeded on plastic, Rab25 and GAPDH protein levels were quantified by Western Blotting. Membranes were imaged with a Licor Odyssey Sa system. Representative of N=2 independent experiments.

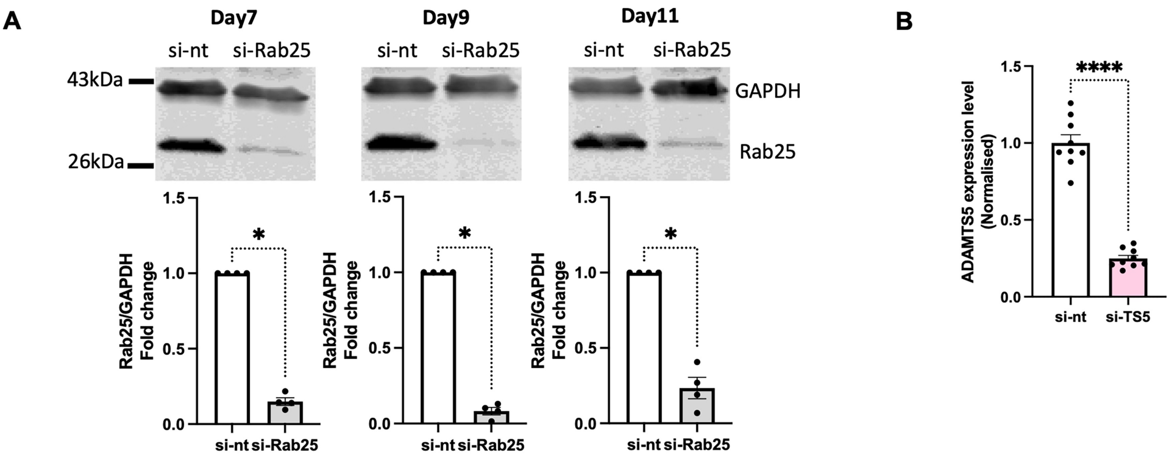

Figure S3. Rab25 and ADAMTS5 KD efficiency in OVCAR3 cells. (A) OVCAR3 cells were transfected with a non-targeting (si-nt) or Rab25-targeting (si-Rab25) siRNA, cell lysates were collected after 7, 9 and 11 days and Rab25 and GAPDH protein levels were quantified by Western Blotting. Membranes were imaged with a Licor Odyssey Sa system, and the band intensity was quantified by Image Studio Lite software. Rab25/GAPDH intensity was normalised to si-nt. Data are presented as mean ± SEM from N=4 independent experiments; * p=0.0286, Mann-Whitney test. **(B)** OVCAR3 cells were transfected with a non-targeting siRNA control (si-nt) or ADAMTS5-targeting (si-TS5) siRNA and the mRNA levels of ADAMTS5 and GAPDH were measured by qPCR. Data were normalised to si-nt and presented as mean ± SEM from N=3 independent experiments. ****p<0.0001, Mann-Whitney test.
